# Oral berberine improves brain dopa/dopamine levels to ameliorate Parkinson’s disease by regulating gut microbiota

**DOI:** 10.1038/s41392-020-00456-5

**Published:** 2021-02-24

**Authors:** Yan Wang, Qian Tong, Shu-Rong Ma, Zhen-Xiong Zhao, Li-Bin Pan, Lin Cong, Pei Han, Ran Peng, Hang Yu, Yuan Lin, Tian-Le Gao, Jia-Wen Shou, Xiao-Yang Li, Xian-Feng Zhang, Zheng-Wei Zhang, Jie Fu, Bao-Ying Wen, Jin-Bo Yu, Xuetao Cao, Jian-Dong Jiang

**Affiliations:** 1 State Key Laboratory of Bioactive Substance and Function of Natural Medicines, Institute of Materia Medica, Chinese Academy of Medical Sciences/Peking Union Medical College, Beijing, 100050 China; 2 The First Hospital of Jilin University, Changchun, 130021 China; 3 Department of Immunology & Centre for Immunotherapy, Institute of Basic Medical Sciences, Peking Union Medical College, Chinese Academy of Medical Sciences, Beijing, 100730 China

**Keywords:** Drug regulation, Drug development, Translational research

## Abstract

The phenylalanine–tyrosine–dopa–dopamine pathway provides dopamine to the brain. In this process, tyrosine hydroxylase (TH) is the rate-limiting enzyme that hydroxylates tyrosine and generates levodopa (l-dopa) with tetrahydrobiopterin (BH_4_) as a coenzyme. Here, we show that oral berberine (BBR) might supply H^•^ through dihydroberberine (reduced BBR produced by bacterial nitroreductase) and promote the production of BH_4_ from dihydrobiopterin; the increased BH_4_ enhances TH activity, which accelerates the production of l-dopa by the gut bacteria. Oral BBR acts in a way similar to vitamins. The l-dopa produced by the intestinal bacteria enters the brain through the circulation and is transformed to dopamine. To verify the gut–brain dialog activated by BBR’s effect, *Enterococcus faecalis* or *Enterococcus faecium* was transplanted into Parkinson’s disease (PD) mice. The bacteria significantly increased brain dopamine and ameliorated PD manifestation in mice; additionally, combination of BBR with bacteria showed better therapeutic effect than that with bacteria alone. Moreover, 2,4,6-trimethyl-pyranylium tetrafluoroborate (TMP-TFB)-derivatized matrix-assisted laser desorption mass spectrometry (MALDI-MS) imaging of dopamine identified elevated striatal dopamine levels in mouse brains with oral *Enterococcus*, and BBR strengthened the imaging intensity of brain dopamine. These results demonstrated that BBR was an agonist of TH in *Enterococcus* and could lead to the production of l-dopa in the gut. Furthermore, a study of 28 patients with hyperlipidemia confirmed that oral BBR increased blood/fecal l-dopa by the intestinal bacteria. Hence, BBR might improve the brain function by upregulating the biosynthesis of l-dopa in the gut microbiota through a vitamin-like effect.

## Introduction

The gut microbiota is an increasingly recognized factor that influences brain function,^1–6^ but the mechanism is unknown. Dopamine is a predominant neurotransmitter, and its level in the brain is closely related to the brain function. Dopamine is primarily synthesized in brain neuron cells.^7^ The major synthetic route for dopamine in humans is the “l-phenylalanine (Phe) → l-tyrosine (Tyr) → (s)-3,4-dihydroxyphenylalanine (levodopa, l-dopa) → dopamine (Phe–Tyr–dopa–dopamine)” metabolic pathway.^8,9^ In this process, the first phase (Phe–Tyr step) is catalyzed by phenylalanine hydroxylase (PAH), and the conversion mainly occurs in liver and kidney.^10^ The liver (or kidney) and/or blood-borne Tyr (from the diet) could cross the blood–brain barrier and enter brain. The Tyr–dopa–dopamine transformation is the second phase and completed in the brain by the enzymes tyrosine hydroxylase (TH) and dopa decarboxylase (DDC).^7,11^ Interruption of this pathway causes neurological diseases, such as phenylketonuria^12^ and Parkinson’s disease (PD).^7,13^

TH is the rate-limiting enzyme for catecholamine biosynthesis which can hydroxylate l*-*Tyr to l-dopa. TH is a type of monooxygenase containing iron, and it needs tetrahydrobiopterin (BH_4_) as a cofactor.^14^ When l-dopa is generated, BH_4_ is transformed into 7,8-dihydrobiopterin (BH_2_), and BH_4_ is consumed continuously in this process. BH_4_ can be synthesized in the cytoplasm of cells in two ways: source synthesis and/or remediation.^15^ Under normal conditions, BH_4_ can be synthesized from the GTP (guanosine triphosphate)–NH_2_P_3_ (D-erythro-7,8-dihydroneopterin triphosphate)–PPH_4_ (6-pyruvoyltetrahydropterin)–BH_4_ pathway, which is called “source synthesis”; on the other hand, BH_4_ can be replenished by means of a remedial pathway of sepiapterin → BH_2_ → BH_4_, and dihydrofolate reductase (DHFR) is the critical enzyme responsible for transforming BH_2_ to BH_4_.^15^ Therefore, discovery of an agonist of DHFR might be an effective way to increase BH_4_ levels and enhance TH activity.

BH_4_-producing bacteria have been evidenced in the intestinal flora,^16^ and the Phe–Tyr–dopa–dopamine metabolic pathway exists in microorganisms as well. This suggests that bacteria may contain homologs of the enzyme genes that mammals utilize to create dopamine.^17–19^ In fact, some of the bacteria could be viewed as a biotech factory to produce dopa/dopamine.^20^ Interestingly, bacteria in the human intestine, such as *Enterococcus*, are producers of dopa/dopamine,^21^ providing a possible chemical link between gut and brain. Recent studies have shown that gut microbiota could modulate brain function through microbial-derived metabolites, such as serotonin.^22^ So, the key question becomes how could we modulate brain function in a practical way.

Berberine (BBR) is a natural compound (Supplementary Fig. S1a, mw 371.8) isolated from herbs, such as *Coptis chinensis* and *Berberis vulgaris*. BBR has been used for decades in China as an over-the-counter (OTC) drug to treat patients with diarrhea. Since 2004, we and others have identified BBR as a safe and efficient medicine for hyperlipidemia and type 2 diabetes with novel mechanisms.^23^ Clinical efficacy of BBR in lowering lipids and glucose has been widely confirmed in the past decade.^23–26^ Furthermore, BBR has been repeatedly reported, from independent groups, to have beneficial effects on brain function.^27,28^ Among the groups, two Korean laboratories investigated the anti-PD effect of BBR in animals. One group found BBR effective in treating PD in mice,^29^ but the other obtained negative results in rats.^30^ In analysis of the difference, we found that the first group gave BBR to mice orally, whereas the second group treated rats with BBR intraperitoneally (i.p.). Thus, we assumed that the gut microbiota might be the answer. In addition, BBR administered through oral route is poorly absorbed in the intestine,^31–33^ and its concentration in intestine is high. This provoked our curiosity to explore the interaction between BBR and the gut flora, as well as the chemical mechanism of BBR that links the gut microbiota with the central nervous system (CNS). The main neurotransmitters, such as l-dopa, have become the research target in this study. We found that oral administration of BBR enhanced TH activity to produce l-dopa by triggering the biosynthesis of BH_4_ in the gut microbiota, elevated blood, and brain dopa/dopamine concentrations, and eventually improved physical performance in animals. Detection of the pathway intermediates in clinical subjects verified the effect of BBR in humans. The results might shed new light on the control of the crosstalk between gut and brain.

## Results

### BBR increased dopa/dopamine production in the gut microbiota in vivo

**Fig. 1:**
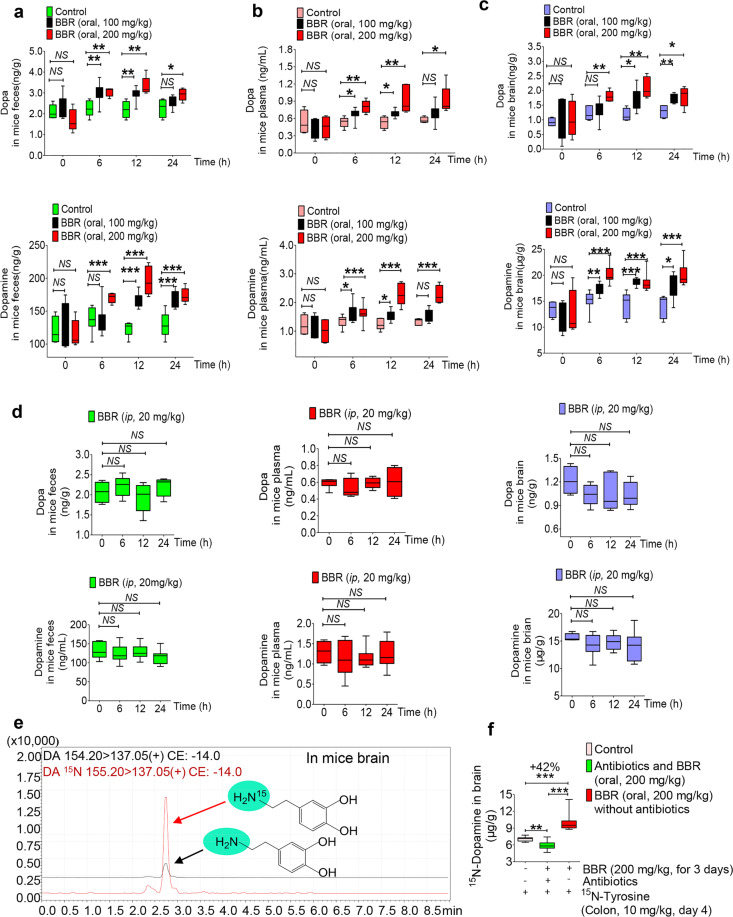
BBR increased dopa/dopamine production in the gut microbiota in vivo. **a** Dopa/dopamine significantly increased in the feces of ICR mice at 0, 6, 12, and 24 h after oral administration of BBR (100 and 200 mg/kg, respectively; mean ± SD, *P < 0.05, **P < 0.01 for dopa and ***P < 0.001 for dopamine). **b** Dopa/dopamine showed a dose- and time-dependent increase in plasma after BBR treatment (mean ± SD, *P < 0.05 and **P < 0.01 for dopa; *P < 0.05, ***P < 0.001 for dopamine). **c** Dopa/dopamine showed a similar dose- and time-dependent increase in the brain after BBR treatment (mean ± SD, *P < 0.05 and **P < 0.01 for dopa; *P < 0.05, **P < 0.01, and ***P < 0.001 for dopamine). **d** Intraperitoneal injection (i.p.) of BBR into the ICR mice did not increase the levels of dopa/dopamine in the feces, plasma, and brain at any of the study time points, which was different from the results of BBR in oral administration (mean ± SD, NS no significance). **e** Mass spectra of ^15^N-dopamine and dopamine in mouse brains. **f** Intestinal ^15^N-Tyr was the raw material for the synthesis of ^15^N-dopamine in brain. Pretreating the mice with antibiotics for 3 days attenuated the effect of BBR on ^15^N-dopamine production (mean ± SD, **P < 0.01, ***P < 0.001), and oral administration of BBR significantly increased ^15^N-dopamine in the brain (*P < 0.05)

L-Dopa is a first-line drug to treat PD, as it could cross the blood–brain barrier and then through the action of DDC be converted into dopamine, which there is a shortage in PD.^34^ Dopa and dopamine (dopa/dopamine) are low-molecular-weight compounds that could be detected in biological samples quantitatively and qualitatively, using liquid chromatography with tandem mass spectrometry (LC-MS/MS; Supplementary Fig. S1b, c). In the present study, we show that oral administration of BBR (100, 200 mg/kg) in mice significantly increased dopa/dopamine production of the intestinal bacteria in 6 h (Fig. 1a). The intestinal dopa/dopamine then entered the blood and brain (dopa only), showing an increased dopa/dopamine in both the blood and brain, in a dose- and time-dependent manner (Fig. 1b, c). The significance of difference seen in dopamine was, in general, larger than that for dopa at the study time points (Fig. 1a), because dopa is unstable and simply converted into dopamine in the presence of tissue DDC.^35^

To verify the role of the gut microbiota in the BBR-induced increase in dopa/ dopamine, pseudo-germ-free (PGF) ICR mice were used with a previously described method.^33^ The mice were pretreated or untreated with oral antibiotics for 3 days, followed by administration of BBR orally with or without concurrent treatment with antibiotics. The intestinal bacterial colony number in the mice treated with antibiotics was significantly lower than that of the mice untreated with antibiotics (−73%, Supplementary Fig. S2a). The increase in dopa/dopamine in feces, blood, and brain was abolished in the BBR-treated mice receiving antibiotics, at all study time points (Supplementary Fig. S2b–d). It appeared that oral antibiotics largely reduced the size of gut flora, and thus decreased their capacity to produce dopa/dopamine in the intestine.

Furthermore, to learn the direct action of BBR in conditions free of intestinal bacteria, ICR mice were treated with BBR via i.p. injection. BBR at 20 mg/kg was used in the i.p. injection experiment, considering that the bioavailability of BBR is much <5%.^31,32^ As shown in Fig. 1d, treating mice with BBR (20 mg/kg, i.p.) caused no change in the level of dopa/dopamine in feces, blood, and brain, suggesting gut microbiota essential for the BBR-induced increase in dopa/dopamine in the body.

In addition, isotope (^15^N)-labeled tyrosine (^15^N-Tyr) was used to validate that the increased brain dopamine by BBR was originated from the intestine. The ^15^N-dopamine and dopamine were identified through comparison of their quantitative ion pair using LC-MS/MS 8050, in which 155.20 → 137.05 (*m*/*z*) was for ^15^N-dopamine and 154.20 → 137.05 (*m*/*z*) was for dopamine. As shown in Fig. 1e, ^15^N-dopamine was detected well in mouse brain, suggesting that the brain ^15^N-dopamine could be from the intestine. Multistage mass spectrometry (LC instrument coupled to an ion trap time-of-flight mass spectrometer, LC/MS^*n*^-IT-TOF) was also used to recognize ^15^N-dopamine and dopamine in brain (Supplementary Fig. S2e). Using dopamine as a reference, ^15^N-dopamine generated a [M + H]^+^ peak at *m*/*z* 155.0861, 1 Da more than dopamine in peak MS^1^; the value of 137.0566 (*m*/*z*) in peak MS^2^ demonstrated a deamination structure, calculated as that of dopamine. The fragments of 119.0476 (*m*/*z*) and 91.0499 (*m*/*z*) from MS^3^ showed values very close to those of dopamine (119.0477 (*m*/*z*) and 91.0499 (*m*/*z*)). The results indicated that dopamine and ^15^N-dopamine could be clearly identified and analyzed quantitatively by LC-MS/MS (Supplementary Fig. S2e). Figure 1f shows that intestinal ^15^N-Tyr was the raw material for the synthesis of ^15^N-dopamine in brain, and oral administration of BBR significantly increased brain ^15^N-dopamine (42%, ***P < 0.001). Pretreating the mice with antibiotics for 3 days (oral, see the “Methods” section) decreased the effect of BBR on ^15^N-dopamine production. The results suggest that the gut microbiota could be a supply for brain dopamine in vivo. Oral antibiotics without BBR treatment has been tested as controls. The result showed that ^15^N-dopamine in brain decreased by −17.2% in the antibiotics treatment group (Supplementary Fig. S2f).

To further verify this hypothesis, blood, liver homogenate, brain homogenate, or gut flora of the ICR mice was incubated with ^15^N-Tyr in vitro, respectively, followed by detection of ^15^N-dopamine generated in the four systems above. The results showed that ^15^N-dopamine was detected in the brain homogenate and gut bacteria (Fig. S2g–j), but not in the blood and liver homogenate, suggesting brain and intestinal bacteria the main sites of dopamine biosynthesis in vivo as they contain both TH and DDC. Furthermore, when treating the brain homogenate or gut bacteria with BBR, the ^15^N-dopamine increased in gut bacteria, but not in the brain because brain tissue does not have dihydroberberine (dhBBR; see content below). It appears that the increased brain dopamine by BBR was from dopa in the gut microbiota, which could be viewed as another “organ” of dopa/dopamine production, in addition to the brain.

### BBR stimulated intestinal bacteria to produce dopa/dopamine in vitro

**Fig. 2:**
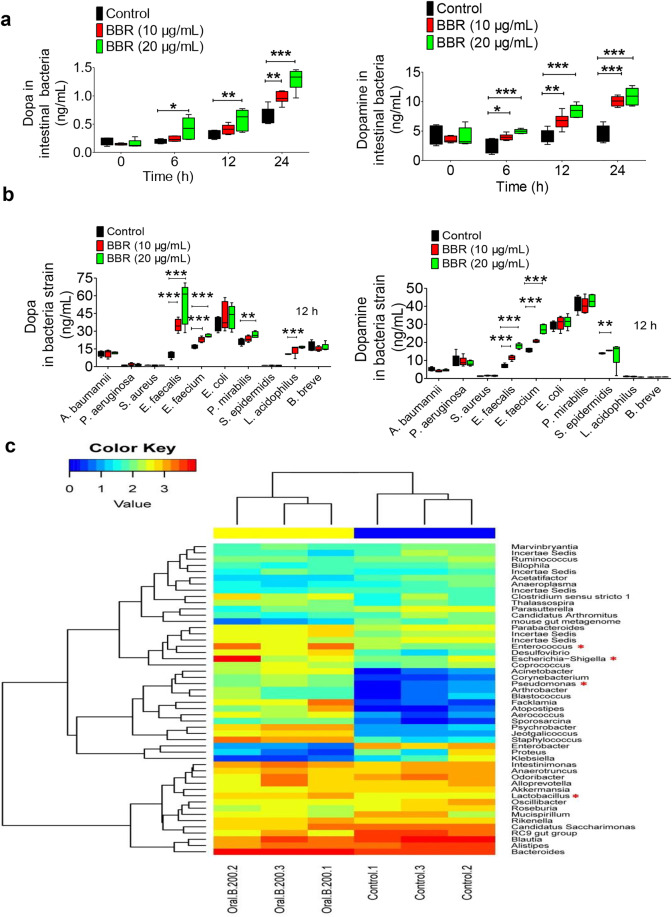
BBR stimulated intestinal bacteria to produce dopa/dopamine. **a** Levels of dopa/dopamine increased significantly at 0, 6, 12, and 24 h after BBR treatment (10 and 20 μg/mL, respectively) in the intestinal bacteria of SD rats in vitro (mean ± SD, *P < 0.05, **P < 0.01, and ***P < 0.001, for dopa and dopamine). **b** Effect of BBR (10 μg/mL) in ten intestinal bacterial strains in vitro. Out of the ten strains, four bacterial strains (*E. faecalis, E. faecium*, *P. mirabilis*, and *L. acidophilus*) showed a significant increase in dopa after BBR treatment (10 and 20 µg/mL for 12 h), six had almost no change in dopa production. Of note, the increase in dopa by *E. faecalis* and *E. faecium* showed a dose-dependent manner with BBR. The BBR-induced dopamine production profile in the ten strains was different from that of dopa, that is, three bacterial strains (*E. faecalis, E. faecium*, and *S. epidermidis*) showed a significant increase in dopamine after BBR treatment (10 µg/mL for 12 h), seven had almost no change on dopamine production. Of note, the increase in dopamine by *E. faecalis* and *E. faecium* showed a similar dose-dependent manner with BBR. **c** Changes in the intestinal bacterial composition as a result of BBR treatment. The heat map of the top 50 bacterial genera exhibited the most substantial change in abundance after exposure to BBR; the proportion of dopa/dopamine-producing bacteria increased, including *Enterococcus*, *Escherichia–Shigella*, *Pseudomonas*, and *Lactobacillus* (labeled with the red “*”)

The experiments were then extended to the in vitro test. Treating intestinal bacteria, as a whole, with BBR (10 and 20 µg/mL) for 24 h in vitro increased their production of dopa/dopamine (Fig. 2a). Then, we detected the effect of BBR on ten intestinal bacterial strains. As shown in Fig. 2b, out of the ten strains, four bacterial strains (*E. faecalis*, *E. faecium, Proteus mirabilis*, and *Lactobacillus acidophilus*) showed a significant increase in dopa after BBR treatment (10 and 20 µg/mL for 12 h), three had almost no change in dopa production, and another three strains had dopa concentrations under the detection level, with or without BBR treatment (not detectable, ND). The dopamine profile by BBR in the ten strains was different from that of dopa, three bacterial strains (*E. faecalis, E. faecium,* and *Staphylococcus epidermidis*) showed a significant increase in dopamine after BBR treatment (10 µg/mL for 12 h), four had almost no change in dopamine production, and another three strains had a dopamine concentration that was ND, with or without BBR treatment. Although there were several strains producing both l-dopa and dopamine, based on their dopa production, we selected *E. faecalis* and *E. faecium* for further investigation. As shown in Supplementary Fig. S3a, b, dopa in both bacterial strains increased 6 and 12 h after BBR treatment (10 µg/mL, *P < 0.05, **P < 0.01), but the increase vanished at 24 h for *E. faecalis*, probably due to the conversion from dopa to dopamine. Meanwhile, the production of dopamine in these two strains significantly increased over time (*P < 0.05, **P < 0.01, and ***P < 0.001 for 6, 12, and 24 h time points). *P. mirabilis* or *Escherichia coli* was not responsive to BBR at all (Supplementary Fig. S3c, d). On the other hand, incubating the tissue homogenate of mouse brain, intestine, or liver, with BBR did not increase the dopa/dopamine levels (Supplementary Fig. S3e–g). The results supported that the gut microbiota is the main target of BBR for dopa/dopamine production in the body.

In addition, changes in the intestinal bacterial composition were detected as well. The ICR mice were treated orally with BBR (200 mg/kg) for 24 h and their fecal samples were taken for bacterial composition analysis. The barcode pyrosequencing of the V3/V4 regions of the 16 S rRNA gene revealed an augmentation of the dopa/dopamine-producing intestinal bacteria in mice treated with BBR. Figure 2c shows the heat map of the top 50 bacterial genera that exhibited the most substantial change in abundance after exposure to BBR. Of the 50 genera, 18 had their abundance increased after BBR treatment, of which 4 genera were documented to be dopa/dopamine producers, including *Enterococcus*,^21^ *Escherichia–Shigella*,^36^ *Pseudomonas*,^37^ and *Lactobacillus*^38^ (Fig. 2c). The increase in the abundance of dopa/dopamine-producing bacteria by BBR might represent a favored selection of BBR in the gut bacterial community. Thus, BBR appeared to elevate dopa/dopamine levels in the gut microbiota by either stimulating dopa/dopamine biosynthesis in bacteria or increasing the abundance of dopa/dopamine-producing bacteria.

### BBR activated dopa/dopamine biosynthesis in gut microbiota and improved brain function in animals

**Fig. 3:**
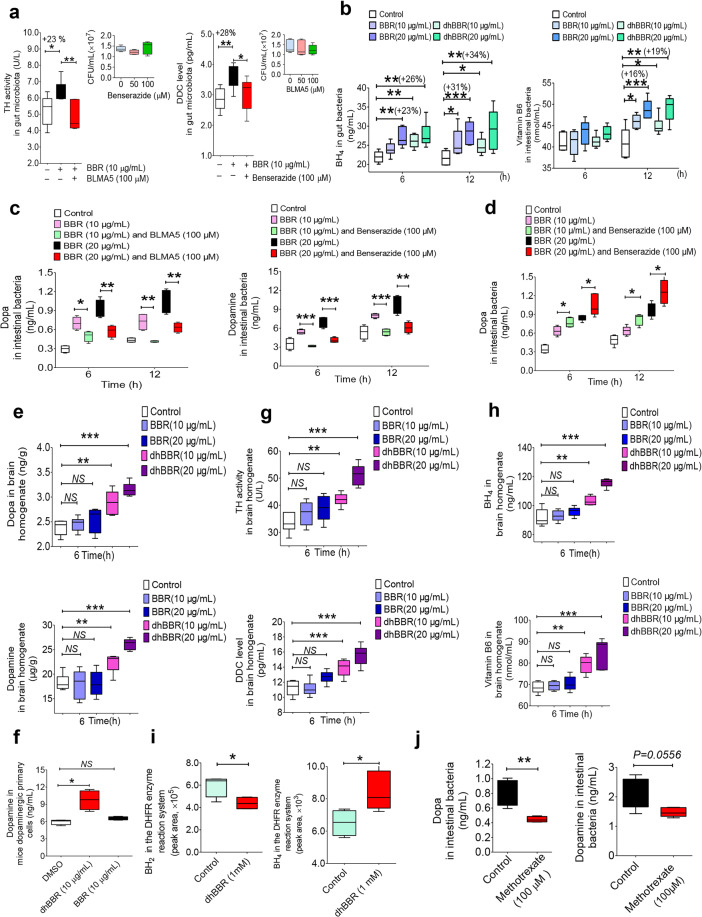
BBR activated the pathway for dopa/dopamine synthesis in the gut microbiota. **a** The activity of TH and DDC increased after treating the gut bacteria of SD rats with BBR at 12 h (10 μg/mL, *P < 0.05, **P < 0.01), but weakened when TH inhibitor (BLMA5, 100 μM) or the DDC inhibitor (benserazide, 100 μM) was incubated with BBR as a whole (mean ± SD, *P < 0.05, **P < 0.01). The TH inhibitor BLMA or DDC inhibitor benserazide did not change the abundance of the intestinal bacteria at 50 or 100 μM. The activity of TH and DDC increased after treating the gut bacteria of SD rats with BBR at 12 h (10 μg/mL, *P < 0.05, **P < 0.01), but weakened when the TH inhibitor (BLMA5, 100 μM) or DDC inhibitor (benserazide, 100 μM) was incubated with BBR as a whole (mean ± SD, *P < 0.05, **P < 0.01). **b** Levels of BH_4_ (the coenzyme of TH) increased significantly at 6 and 12 h after BBR or dhBBR treatment (10 and 20 μg/mL, respectively) in SD gut bacteria in vitro (mean ± SD, +23%↑, +31%↑ for BBR at 20 μg/mL at 6 and 12 h, **P < 0.01, ***P < 0.001; +26%↑, +34%↑ for dhBBR at 20 μg/mL at 6 and 12 h, **P < 0.01). In addition, the level of DDC coenzyme VB_6_ was enhanced in the intestinal bacteria at 12 h (mean ± SD, +16%↑ for BBR at 20 μg/mL, ***P < 0.001; +19%↑ for dhBBR at 20 μg/mL, **P < 0.01). **c** The TH inhibitor (BLMA5, 100 μM) decreased the production of dopa in SD rat gut bacteria when treated with BBR for 6 and 12 h (*P < 0.05, **P < 0.01); DDC inhibitor (benserazide, 100 μM) also lowered the dopamine generation significantly when incubated with BBR (mean ± SD, **P < 0.01, ***P < 0.001). **d** The DDC inhibitor (benserazide, 100 μM) increased the generation of dopa in SD rat gut bacteria when treated with BBR (mean ± SD, *P < 0.05). **e** Levels of dopa/dopamine increased significantly at 6 hr after dhBBR treatment in the brain homogenate of mice in vitro (mean ± SD, **P < 0.01, ***P < 0.001). **f** Coincubation of mouse dopaminergic primary cells with BBR or dhBBR (10 μg/mL), significantly increased dopamine levels in the dopaminergic primary cells incubated with dhBBR (*P < 0.05). **g** Levels of TH/DDC increased significantly at 6 h after dhBBR treatment in the brain homogenate of mice in vitro (mean ± SD, **P < 0.01, ***P < 0.001). **h** Levels of BH_4_/VB_6_ increased significantly at 6 h after dhBBR treatment in the brain homogenate of mice in vitro (mean ± SD, **P < 0.01, ***P < 0.001). **i** BH_2_ decreased significantly (*P < 0.05) with increasing BH_4_ (*P < 0.05), when dhBBR was added to the mixture of BH_2_ (as substrate) and dihydrobiopterin reductase at 37 °C for 2 h. **j** Levels of dopa/dopamine decreased significantly at 12 h after the treatment with DHFR inhibitor (methotrexate 100 μM) in the intestinal bacteria (mean ± SD, *P < 0.05)

TH and DDC are two key enzymes that respectively convert tyrosine to dopa and then dopa to dopamine.^39^ We first determined the activity of TH and level of DDC after exposing the bacteria to BBR for 12 h. Enzyme assays showed that BBR (10 μg/mL) treatment in vitro increased the TH activity in intestinal flora by 23% and DDC level by 28% (Fig. 3a, *P < 0.05, **P < 0.01). Meanwhile, we first measured the influence of BLMA5 and benserazide on enzyme activity or level of TH and DDC. The results showed that the activity of TH or level of DDC was significantly inhibited by TH or DDC inhibitors respectively (**P < 0.01 for TH; *P < 0.05 for DDC, Supplementary Fig. S3h). As the coenzyme of TH, the BH_4_ level was enhanced in the intestinal bacteria after the treatment with BBR or its intestinal metabolite dhBBR at 6 and 12 h (Fig. 3b, +23%↑, +31%↑ for BBR at 20 μg/mL, **P < 0.01, ***P < 0.001; +26%↑, +34%↑ for dhBBR at 20 μg/mL, **P < 0.01), with the chemical mechanism illustrated below. In addition, the DDC coenzyme vitamin B6 (VB_6_) level was enhanced in the intestinal bacteria at 12 h (Fig. 3b, +16%↑ for a BBR dose of 20 μg/mL, ***P < 0.001; +19%↑ for a dhBBR dose of 20 μg/mL, **P < 0.01). We consider it might be the main mechanism by which BBR promotes dopa/dopamine production in bacteria. If true, inhibition of the two enzymes might suppress BBR-induced dopa/dopamine production in the gut microbiota. First, we showed that TH inhibitor bleomycin A5 (BLMA5) or DDC inhibitor benserazide did not change the abundance of the intestinal bacteria in the range of 0–100 μM (Fig. 3a). Next, as shown in Fig. 3c, the addition of TH inhibitor BLMA largely reduced BBR’s effect (10 or 20 µg/mL, for 6 or 12 h) on dopa production in the gut microbiota (**P < 0.01), and the addition of DDC inhibitor benserazide (Fig. 3c) reduced the BBR-induced production of dopamine (***P < 0.001). In addition, we treated the gut microbiota with BBR (10 or 20 µg/mL for 6 or 12 h) in combination with benserazide. As shown in Fig. 3d, combination of BBR with benserazide further increased the dopa level in the gut microbiota, most likely because the conversion from dopa to dopamine was blocked, while dopa biosynthesis was increased by BBR. The results revealed a stimulatory activity of BBR on dopa/dopamine biosynthesis, working through activation of both TH and DDC in the gut microbiota. As intestinal dopamine could not cross the blood–brain barrier, our focus was on dopa. Combination of BBR with DDC inhibitors should further increase dopa in blood and therefore the level of dopamine in brain.

**Fig. 4:**
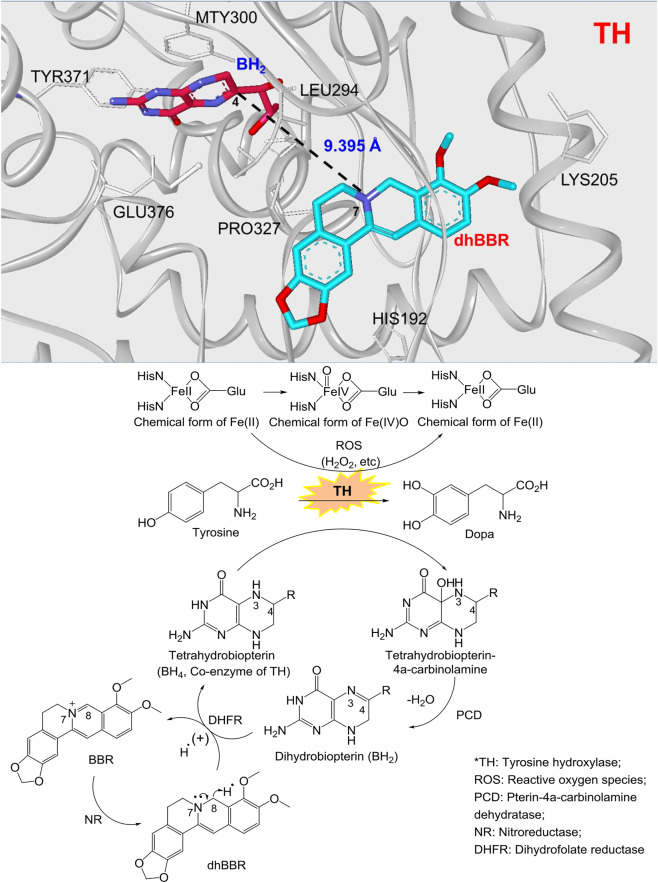
The dhBBR-mediated chemical reaction for the conversion from tyrosine to dopa. BH_2_ obtained H^•^ from the dhBBR–BBR system, and its reduction was activated, in which H^•^ from dhBBR (N_7_–C_8_) moved to the N_3_=C_4_ bond in BH_2_, and N_3_=C_4_ was then transformed into N_3_–C_4_ (BH_2_ was converted into BH_4_). In the meanwhile, dhBBR lost H^•^, and was oxidized into BBR. The increased BH_4_ in intestinal bacteria accelerates the transformation from tyrosine to l-dopa, in the presence of ROS (H_2_O_2_, etc.) as well as Fe^2+^. Accordingly, BH_4_ is converted into tetrahydrobiopterin–4α-carbinolamine

Although TH and DDC are present in the brain, coincubation of brain homogenate with BBR did not increase the brain levels of dopa/dopamine (Fig. 3e). However, coincubation of brain homogenate with dhBBR, a BBR metabolite produced by nitroreductase in gut flora, significantly increased the dopa/dopamine in the brain (Fig. 3e, **P < 0.01, ***P < 0.001; Fig. 3f, *P < 0.05). Accordingly, we found that the activity of TH (and the coenzyme BH_4_) and the level of DDC (and the coenzyme VB_6_) in brain was elevated by dhBBR (Fig. 3g, h). Thus, BBR-induced increase in dopa/dopamine production in the gut microbiota might be mediated through its intestinal metabolite dhBBR, which could activate TH and DDC by elevating BH_4_ and VB_6_ levels, respectively. The putative chemical mechanism of dhBBR on TH activity is shown in Fig. 4.

DhBBR exhibited excellent docking performance in docking onto TH (pdb: 2TOH) with a binding free energy of –44.26 kcal/mol. DhBBR anchored into the binding site of TH through multiple interactions with its scaffold and the side chains (Fig. 4). This binding pocket is also the one for BH_2_. The distance between the 7-N atom of the N–C group in dhBBR and the C_4_ atom of the N_3_=C_4_ bond of BH_2_ was ~9.395 Å, which is within the optimal distance for electron transfer. When dhBBR approached BH_2_ in the pocket, BH_2_ obtained H^**•**^ from the dhBBR–BBR system, and its reduction was activated in which H^**•**^ from dhBBR (N_7_–C_8_) moved to the N_3_=C_4_ bond in BH_2_, and N_3_=C_4_ was then transformed into N_3_–C_4_ (BH_2_ was converted into BH_4_). In the meanwhile, dhBBR lost H^**•**^ and was oxidized into BBR (Fig. 4). The increased BH_4_ in intestinal bacteria accelerated the transformation from tyrosine to l-dopa in the presence of reactive oxygen species (ROS; H_2_O_2_, etc.), as well as Fe^2+^. Accordingly, BH_4_ is converted into tetrahydrobiopterin–4α-carbinolamine. The chemical mechanism is shown in Fig. 4.^33,40–42^ To verify that dhBBR activates the TH enzyme by promoting BH_4_, we added dhBBR to the mixture of BH_2_ (as substrate) and DHFR at 37 °C for 2 h. The results showed that BH_2_ decreased significantly (*P < 0.05) accompanied by increasing BH_4_ (*P < 0.05; Fig. 3i), suggesting that dhBBR might promote biotransformation from BH_2_ to BH_4_ in the bioactivity of DHFR. Besides, when the inhibitor of DHFR (methotrexate, 100 μM) was added, the production of dopa/dopamine was significantly inhibited (**P < 0.01 for dopa; *P* = 0.0556 for dopamine, shown in Fig. 3j). It appeared that dhBBR is an agonist of DHFR possessing a similar function to that of the coenzyme NADPH.

Since brain tissue is free of nitroreductase and does not have dhBBR,^33^ the dopa/dopamine production in the brain tissue treated with BBR remains unchanged.^33^ In fact, the in vivo experiments showed that dhBBR was detectable in the intestine only, but not in the blood and brain,^33^ strongly supporting the assumption that the increased dopamine in the brain by BBR was from the gut microbiota.

**Fig. 5:**
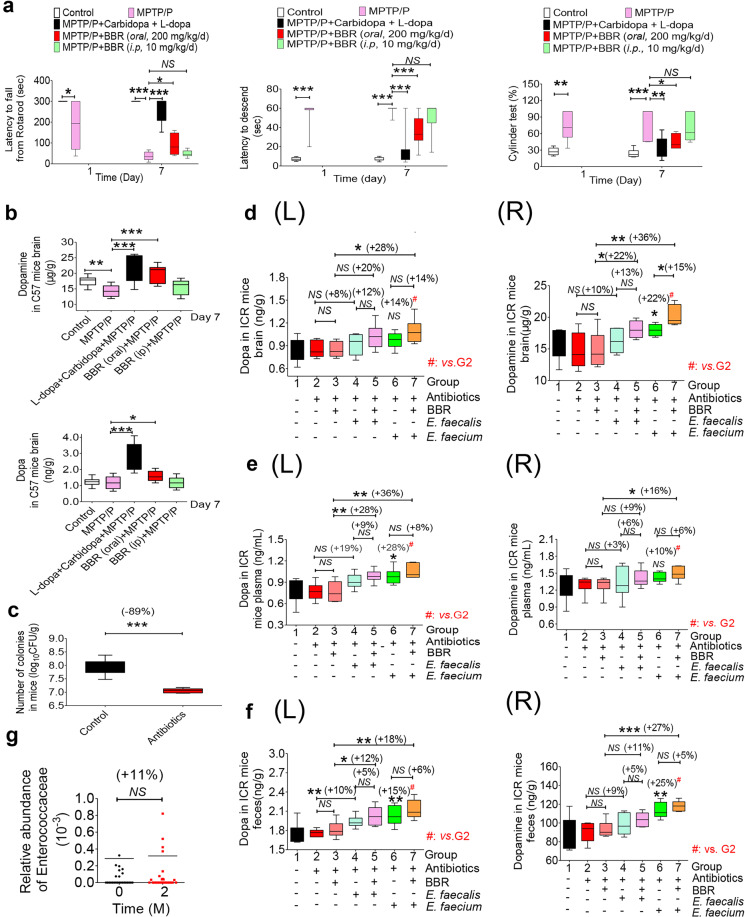
*E*. faecalis and *E*. faecium regulated dopa/dopamine and improved brain function in PD animals in the presence of BBR. **a** Treating mice with both MPTP (20 mg/kg/d, s.c.) and P (200 mg/kg/d, i.p.) for 7 days successfully generated the damage to brain function (LD, ***P < 0.001; LFR, ***P < 0.001) in the model mice. Carbidopa (1 mg/kg, s.c.) in combination with l-dopa (10 mg/kg/d, s.c.) for 6 days (from days 2 to 7) decreased the LD, cylinder test value (***P < 0.001, **P < 0.01), and increased the LFR (***P < 0.001); BBR (200 mg/kg/d, orally, days 2 to 7) significantly reduced the LD, cylinder test value (***P < 0.001, *P < 0.05), and elevated the LFR (*P < 0.05). Intravenous injection of BBR (10 mg/kg, for 6 days) did not cause a significant change in the LD, LFR, or cylinder test. **b** Levels of dopa/dopamine in the brain on day 7 of the experiment (mean ± SD, *P < 0.05 for dopa; **P < 0.01, ***P < 0.001 for dopamine). **c** Number of colonies from the mice treated with the antibiotics was significantly lower than that from the normal mice (mean ± SD, ***P < 0.001). **d**–**f** Translation of *E. faecalis* and *E. faecium* into the mice treated with antibiotics significantly increased dopa (L)/dopamine (R) levels in ICR mouse brains (**d**, *P < 0.05, **P < 0.01, NS no significance), blood (**e**, *P < 0.05, **P < 0.01), and feces (**f**, (L), (R), *P < 0.05, **P < 0.01, ***P < 0.001). **g** The relative abundance of Enterococcaeae in the fecal samples of clinical subjects increased after the two-month BBR treatment. Data are represented as the mean ± SD

To learn whether the BBR-induced increase in dopa/dopamine could improve physical performance in animals, a brain functional test was performed in PD mouse models using established methods described previously.^43^ As shown in Fig. 5a, b, treating mice with both 1-methyl-4-phenyl-1,2,3,6-tetrahydropyridine hydrochloride (MPTP-HCL, 20 mg/kg/d, s.c.) and probenecid (P, 200 mg/kg/d, i.p.) for 7 days successfully generated damage in brain function, as demonstrated by a significant increase in latency to descent (LD, from 8 to 60 s, ***P < 0.001), a decrease in latency to fall from rotarods (LFR, from 300 to 38 s, ***P < 0.001), and an increase by the ratio of ipsilateral touches to the total of ipsilateral and contralateral touches on the wall (separately counted) in the cylinder test (CT, from 27 to 72%, **P < 0.01; Fig. 5a) in the disease model mice. Treating the model mice with carbidopa (1 mg/kg, s.c.) in combination with l-dopa (10 mg/kg/d, s.c.) for 6 days (from days 2 to 7) decreased the time of LD from 60 to 18 s (**P < 0.01), increased the LFR from 38 to 256 s (***P < 0.001), as well as decreased the ratio of CT from 72 to 35% (**P < 0.01), demonstrating a significant therapeutic effect of the l-dopa treatment. Treating the model mice orally with BBR (200 mg/kg/d) for 6 days (from days 2 to 7) significantly reduced the LD from 60 to 36 s (***P < 0.001), elevated the LFR from 38 to 92 s (*P < 0.05), and decreased the ratio in CT from 72 to 45% (*P < 0.05). I.p. injection of BBR (10 mg/kg, for 6 days) caused no significant change in the LD, LFR, or CT in the model mice. Chemical analysis showed that by day 7 of the experiment, dopa/dopamine in brain tissue was significantly higher in the BBR oral treatment group than that in the disease model mice (*P < 0.05 for dopa, ***P < 0.001 for dopamine, Fig. 5b). The increase in dopa in the brains of mice treated with BBR was less significant than that of those treated directly with l-dopa plus carbidopa (Fig. 5b). It appears that in these mouse models, BBR partially ameliorated brain functional damage, and the elevation of dopa/dopamine production in the gut microbiota could be at least part of the mechanism. In this experiment, l-dopa treatment showed a therapeutic efficacy that was better than that of BBR, probably because of its combination use with carbidopa, a DDC inhibitor.

### Transplantation of *E. faecalis* and *E. faecium* in animals increased brain dopa/dopamine and improved brain function

First, the enzymatic activity of TH and DDC level in the *E. faecalis* and *E. faecium* strains was detected. Considerable enzymatic activity of TH or DDC level was detected in both *E. faecalis* and *E. faecium*, in which the TH activity was 1.66 or 1.40 mU/10^8^ colony forming units (CFU) and DDC level was 0.65 or 0.62 pg/10^8^ CFU, respectively. No significant difference of the enzyme assay was observed between the two bacteria (Fig. S3i). Indeed, the two bacteria synthesized dopa/dopamine and the production was enhanced by BBR (Supplementary Fig. S3a, b).

We then transplanted *E. faecalis* and *E. faecium* into the PGF mice pretreated with antibiotics. Their intestinal bacterial colony number was decreased by 89% on average, which was significantly lower than that of the untreated mice (***P < 0.001, Fig. 5c).

Then, the mice were randomly divided into seven groups (*n* = 6 per group). Normal mice treated with PBS were in group 1, as control. Group 2 was treated with antibiotics only as the PGF reference (control); group 3 was treated with BBR (200 mg/kg/d, oral); group 4 was treated with *E. faecalis* (6 × 10^8^ CFU/d, oral) alone; group 5 was treated with *E. faecalis* and BBR (200 mg/kg/d, oral); group 6 was treated with *E. faecium* (6 × 10^8^ CFU/d, oral) alone; and group 7 was treated with *E. faecium* and BBR (200 mg/kg/d, oral). The mice in the groups 2–7 were orally given antibiotics (see the “Method” section) twice a day for 3 days in advance, and then BBR (200 mg/kg) and/or bacteria were orally given to five of the groups (groups 3–7) for successive 3 days. Three days after bacterial transplantation, the brain, blood, and feces were collected for dopa and dopamine measurement. We should mention here that in the early experiments of bacterial transplantation, we found that the transplanted bacteria had successfully colonized in mouse intestine, for example, in *E. faecium* transplantation (*P < 0.05; Fig. S3j), ensuring the prerequisite of the experiment.

As shown in Fig. 5d–f, 3 days after *E. faecalis* and *E. faecium* transplantation, the levels of dopa/dopamine in brain, blood, and feces of the PGF mice were considerably increased, compared to those in the PGF mice (group 2). The mice treated with *E. faecium* showed an elevation of 14% in brain dopa (group 6 vs. group 2, in Fig. 5d(L)) and a significant increase of 22% in brain dopamine (group 6 vs. group 2, in Fig. 5d(R), *P < 0.05). In addition, dopa in blood increased by 28% (group 6 vs. group 2, in Fig. 5e(L), **P < 0.01), dopamine increased by 10% (group 6 vs. group 2, in Fig. 5e(R)), dopa in feces increased by 15% (group 6 vs. group 2, in Fig. 5f(L), **P < 0.01), and dopamine increased by 25% (group 6 vs. group 2, in Fig. 5f(R), **P < 0.01). For the mice with *E. faecalis*, the levels of dopa/dopamine were also elevated in the brain, blood, and feces of the mice (group 4 vs. group 2, Fig. 5d–f), but not as high as those with *E. faecium*, suggesting that the ability of synthesizing dopa/dopamine in *E. faecium* was slightly stronger than that of *E. faecalis* (group 6 vs. group 4, in Fig. 5d–f). In additionally, *E. coli* was transplanted into the PGF mice for comparison. As shown in Figs. S4a–c, 3 days after *E. coli* transplantation, the levels of dopa/dopamine in brain, blood, and feces of the PGF mice remained unchanged, while the mice treated with *E. faecium* and *E. faecalis* showed a significant increase in dopa/dopamine in the brain, consistent with the in vitro results (in Fig. S3a, b). Considering that TH-mediated biochemical reactions require the presence of ROS,^44^ ROS in the intestinal bacteria was measured. The results showed that ROS were detectable in *E. coli*, *E. faecium*, and *E. faecalis*, with which TH could convert tyrosine into l-dopa (*P < 0.05, **P < 0.01, and ***P < 0.001; Fig. S3k).

BBR treatment did not increase the levels of dopa/dopamine in the PGF mice (group 3 vs. group 2, NS, Fig. 5d–f), but promoted either *E. faecalis* or *E. faecium* to synthesize dopa/dopamine in the PGF mice. As shown in Fig. 5d(R), *E. faecium* combined with BBR increased brain dopamine by 15% (group 7 vs. group 6, *P < 0.05) compared with *E. faecium* alone, which may be due to the stimulation effect of BBR on the intestinal bacteria TH and DDC. In addition, combination of BBR with *E. faecium* increased brain dopamine by 36% (group 7 vs. group 3, **P < 0.01, Fig. 5d(R)) and brain dopa by 28% (group 7 vs. group 3, **P < 0.05, Fig. 5d(L)) compared with those in the PGF mice with BBR alone. BBR plus *E. faecalis* also resulted in an increase in brain dopamine by 22% (group 5 vs. group 3, Fig. 5d(R), *P < 0.05) and brain dopa by 20% (group 5 vs. group 3, Fig. 5d(L)). Levels of dopa/dopamine in plasma or feces showed an escalation profile similar to that in brain (Fig. 5e, f; group 5 vs. group 3, group 7 vs. group 3, **P < 0.01 and *P < 0.05, respectively).

As we have seen in Fig. 5d–f, treatment with antibiotics could reduce the bacterial colony in the mouse intestine; however, dopa and dopamine in the feces, plasma, and brain of the PGF mice did not change significantly. The explanation could be first that gut microbiota might not be the major contributor for brain dopamine in nature; thus, dopamine level in brain and blood is relatively stable, even the bacterial colony numbers decreased; and second, *Enterococcus* appeared relatively more resistant to the antibiotics as compared with other bacteria in intestine (Supplementary Fig. S3j), thus the residual *Enterococcus* remains good production of dopa/dopamine. Other reasons might not be excluded. Actually, dopamine level in brain was decreased by antibiotics ~10% evidenced in the brain dopamine test (Fig. 5d(R), −7.8%), as well as in the ^15^N-labeled experiment (Supplementary Fig. S2f, −17.2%).

These results suggested that *E. faecalis* or *E. faecium* might play a key role in synthesizing dopa and dopamine if transplanted into the body; BBR treatment could activate the TH and DDC enzymes of the bacteria and enhance dopa/dopamine production in the intestine, eventually resulting in elevated dopa/dopamine in the brain. Accordingly, the behavioral improvement was observed in the PD model mice treated with the bacteria and BBR (Supplementary Fig. S5a, b and S6a–c).

### 2,4,6-Trimethyl-pyranylium tetrafluoroborate-derivatized matrix-assisted laser desorption mass spectrometry imaging of dopamine in ICR mouse brains

To further demonstrate that the interaction between BBR and *E. faecalis/E. faecium* benefits dopamine generation, we employed TMP-TFB-derivatized MALDI-MS imaging method to investigate the distribution of striatal dopamine in each group.^45^ Eighteen ICR mice (male, 18–22 g) were randomly divided into six groups, namely groups 1–6. The mice in groups 1 and 2 were treated with PBS, and the mice in group 3 were treated with *E. faecalis* (6 × 10^8^ CFU/d, oral); the mice in group 4 were treated with *E. faecalis* and BBR (200 mg/kg/d, oral); the mice in group 5 were treated with *E. faecium* (6 × 10^8^ CFU/d, oral); and the mice in group 6 were treated with *E. faecium* and BBR (200 mg/kg/d, oral). The mice in groups 2–6 were orally given cefadroxil (100 mg/kg), terramycin (300 mg/kg), and erythromycin (300 mg/kg) twice a day for 3 days, and then BBR or bacteria was orally given to the four groups for three successive days.

**Fig. 6:**
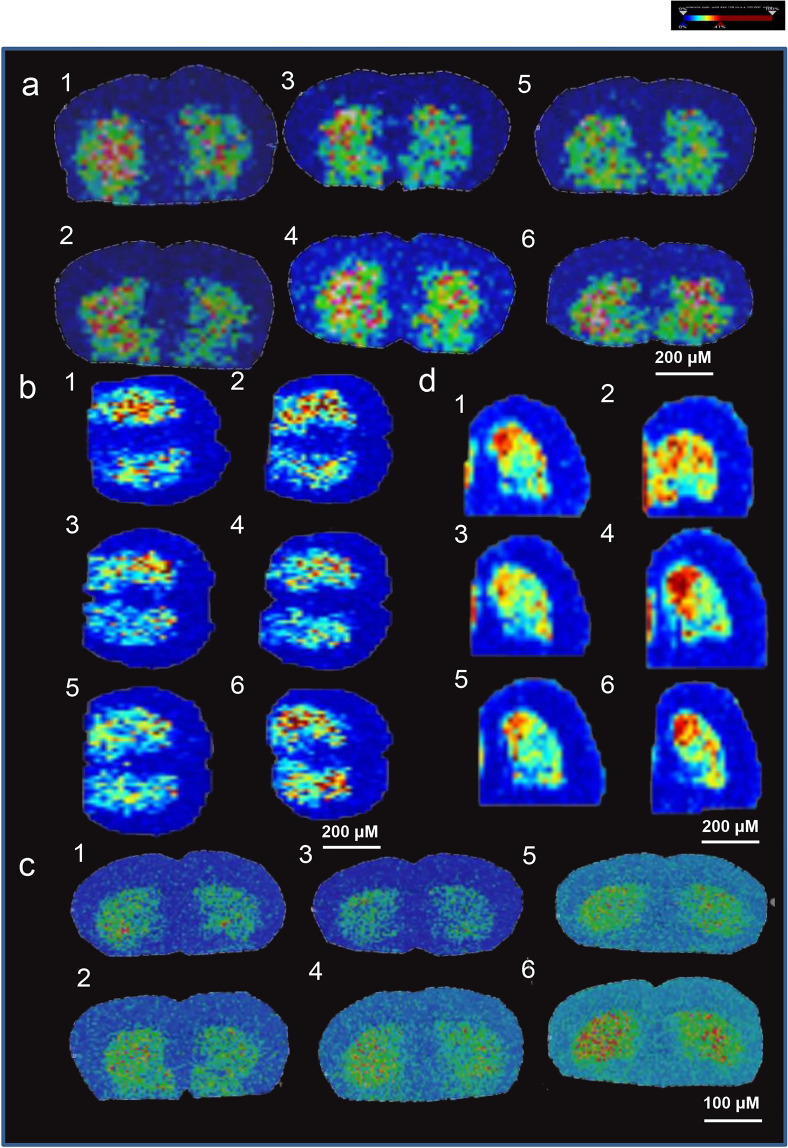
BBR stimulated the production of dopamine in striatum in mice transplanted with *E*. faecalis or *E*. faecium: TMP-TFB-derivatized MALDI-MS images. **a** The relative abundance and distributions of dopamine with a spatial resolution of 200 µm. **b** Rescaled image from **a** with baseline subtraction and total ion count normalization. **c** The relative abundance and distributions of dopamine with a spatial resolution of 100 µm. **d** The relative abundance and distributions of dopamine in half brains from the same mice in **c** with a spatial resolution of 200 µm. All of them showed elevated levels of dopamine after BBR addition. (group 1: normal group; group 2: antibiotic treated only; group 3: antibiotics and *E. faecalis*; group 4: antibiotics, *E. faecalis*, and BBR (200 mg/kg); group 5: antibiotics, *E. faecium*; and group 6: antibiotics, *E. faecium*, and BBR (200 mg/kg)). Blue represents the decreased level and red represents the increased level. The striatal dopamine level in the mice receiving both *E. faecalis* and BBR was higher than that treated with *E. faecalis* only (group 3 vs. group 4). The striatal dopamine in the mice receiving both *E. faecium* and BBR was higher than that treated with *E. faecium* only (group 5 vs. group 6)

As shown in Fig. 6, the results are presented in four different ways, including the relative abundance and distributions of dopamine with a spatial resolution of 200 µm (Fig. 6a); rescaled image from Fig. 6a with baseline subtraction and total ion count normalization (Fig. 6b); the relative abundance and distributions of dopamine with a spatial resolution of 100 µm (Fig. 6c); and the relative abundance and distributions of dopamine in half the brain from the same mice in Fig. 6c with a spatial resolution of 200 µm (Fig. 6d). Compared to groups 3 and 5, in which the mice were only treated with bacteria, the striatal dopamine levels in the mice that received both bacteria, and BBR were much higher from the perspective of both the whole brain and half brain (Fig. 6a–d). This result is consistent with what we obtained with brain homogenate (Fig. 5e(R)), which demonstrated that BBR might have the potential to stimulate the dopa/dopamine pathways of *E. faecalis* and *E. faecium* by increasing BH_4_ levels in the intestine. This interaction eventually leads to an elevated amount of dopamine in the brain, of which combination of BBR with *E. faecalis* or *E. faecium* showed an elevation higher than that treated with *E. faecalis* or *E. faecium* alone (Fig. 6a–d).

### BBR increased blood dopa/dopamine in clinical subjects

To learn whether BBR could increase blood dopa in humans, 28 individuals with hyperlipidemia were randomly enrolled in the BBR treatment study in the Outpatient Section of the First Hospital of Jilin University in Changchun in the early spring of 2017 (Clinical approval number ChiCTR-OPN-17012942).

As the first step of the trial, blood, and fecal samples of the 28 subjects were taken for the baseline. Then, BBR (0.5 g, bid) was orally given to the subjects for 8 weeks, followed by sample collection (blood and fecal samples). As shown in Table 1, after 8 weeks of BBR therapy, blood dopa/dopamine was elevated (dopa, NS, +17%↑; dopamine, *P < 0.05, +24%↑). Fecal tests demonstrated results similar to those of blood, showing an increase in dopa and dopamine in feces (dopa, ***P < 0.001, +46%↑; dopamine, ***P < 0.001, +47%↑, Table 1). Meanwhile, TH and DDC in gut microbiota were upregulated by BBR (TH, **P < 0.001, +42%↑; DDC, NS, +20%↑, Table 1). The results agreed with those detected in the animal experiments. Analysis of the microbial diversity in the fecal samples of 28 clinical patients showed that, after 2 months of BBR treatment, the relative abundance of *Enterococcus* increased by 11% (Fig. 5g; Table 1), in which *E. faecalis* and *E. faecium* were predominant.^46^ The results further validated that *Enterococcus* might be an interesting genus for dopa/dopamine biosynthesis in the intestine, and BBR might promote the body dopa/dopamine levels through the bacteria in intestine.

**Table 1:** Berberine regulated the biomarkers in blood and feces of clinical subjects^a^

Biomarker	In blood (mean ± SD, *n* = 28)		
	Untreated^b^	Treated with BBR^c^	(c–b)/b × 100%
Dopa (ng/mL)	1.10 ± 0.45	1.29 ± 0.36^ns^	+17%↑
Dopamine (ng/mL)	1.40 ± 0.48	1.73 ± 0.67*	+24%↑

Biomarker	In feces (mean ± SD, *n* = 28)		
	Untreated^d^	Treated with BBR^e^	(e–d)/d × 100%
Dopa (ng/mL)	1.64 ± 0.48	2.39 ± 0.74***	+46%↑
Dopamine (ng/mL)	80.47 ± 21.43	118.51 ± 39.75***	+47%↑
TH (U/L)	7.07 ± 1.74	10.05 ± 4.05**	+42%↑
DDC (pg/g)	1.88 ± 0.31	2.25 ± 0.90^ns^	+20%↑
Relative abundance of Enterococcaceae (%)	0.0286 ± 0.0133^f^	0.0318 ± 0.0178^f^	+11%↑

*ns* no significant

*P < 0.05, **P < 0.01, ***P < 0.001

^a^Clinical subjects: oral, 1 g/d, for 60 days; data are shown as mean ± SD. ^b^Biomarker levels in blood of untreated subjects. ^c^Biomarker levels in blood of sujects treated with BBR. ^d^Biomarker levels in feces of untreated subjects. ^e^Biomarker levels in feces of subjects treated with BBR

^f^Data are shown as mean ± SEM

## Discussion

BBR is an alkaloid that is poorly absorbed in the intestine,^31–33,47^ but it has been well documented by independent groups that oral administration of BBR showed therapeutic effects on energy metabolism and brain function in patients, as well as in animals,^48^ leaving us with a very interesting question in pharmacological research. We have recently discovered that BBR could stimulate synthetic pathways in gut microbiota to produce short-chain fatty acids, such as acetate, propionate, and butyrate, which steadily enter the blood and lower blood lipids and sugar.^49^ In continuing the searching for BBR-induced bioactive metabolites from gut microbiota, we discovered that the concentrations of Tyr, dopa, and dopamine significantly increased in the intestinal bacteria after exposure to BBR, while Phe decreased. The activity of TH and DDC in gut flora might play an important role in the mode of action of BBR. The key chemical mechanism appears to be associated with the intestinal dhBBR, which accelerates the transformation from BH_2_ to BH_4_ by contributing H^**•**^, leading to an increase in BH_4_ levels and then TH activity.

BH_4_ is a coenzyme of either TH or PAH acting as an electron carrier in enzymatic reactions as a reducing agent.^11^ The oxidation form of BH_4_ is BH_2_, and BH_4_ can be regenerated by NADPH as a hydrogen donor under the catalysis of DHFR.^11^ A deficiency of BH_4_ or the reductase will directly inhibit the hydroxylation of Phe to Tyr and/or Tyr to dopa, which will block the Phe–Tyr–dopa–dopamine metabolism pathway and lead to nervous system diseases.^50^ At present, the main clinical treatment strategy for BH_4_ deficiency is to increase the intake of synthetic BH_4_ (trade name Kuvan).^51^ Here, BBR might be the first agonist of *Enterococcus* bacteria that leads to the production of l-dopa by triggering BH_4_ formation in the gut bacteria. Interestingly, BBR acts in a way similar to vitamins. The molecular details are illustrated in Fig. 4.

Furthermore, BBR could promote dopa/dopamine production through improving the Phe–Tyr–dopa–dopamine synthetic pathway in gut flora, but not in other tissues (liver, small intestine, and brain; Fig. 3e and Supplementary Fig. S3e–g), suggesting that the active compound for dopa/dopamine production is dhBBR, which is converted from BBR by nitroreductase in the intestinal bacteria^33^ and is almost undetectable in blood. Indeed, dopa/dopamine in brain tissue was increased by dhBBR, but not BBR (Fig. 3e). Biochemical studies of the intestinal bacteria revealed that BBR/dhBBR activated bacterial TH, DDC enzymes through their cofactors BH_4_ and VB_6_ (Fig. 3b, g, h), thus efficiently metabolizing dietary Phe into Tyr, and then dopa/dopamine in intestinal cavity (Table 1).

In addition, the proportion of dopa/dopamine-producing bacteria in the gut flora community was also increased after exposure to BBR, indicating a favorable selection of BBR in the gut flora community. BBR-induced intestinal Tyr, dopa, and dopamine entered the blood and eventually showed an increased concentration of dopamine in the brain. Accordingly, brain function in mice injured by injection of MPTP and P was in part protected by BBR oral treatment, at least partially due to the increase in dopamine in the brain. The increased dopa and dopamine levels were further detected in the blood of patients who took BBR for 2 months. The clinical study was performed in patients with hyperlipidemia, as it has been reported that patients with metabolic disorders have a higher incidence of developing neurodegenerative diseases, as compared to those with normal metabolic profiles.^52–55^ The clinical results showed an improved blood level of dopa/dopamine by BBR, of which dopa could enter the brain, be converted into dopamine and improve brain function. To the best of our knowledge, this is the first report to show that regulation of the Phe–Tyr–dopa–dopamine pathway axis in the gut microbiota by a drug could improve brain function. The pathway intermediates (especially dopa) might be the chemical link for the crosstalk between gut and brain (Figs. 4 and and 7).^56^

**Fig. 7:**
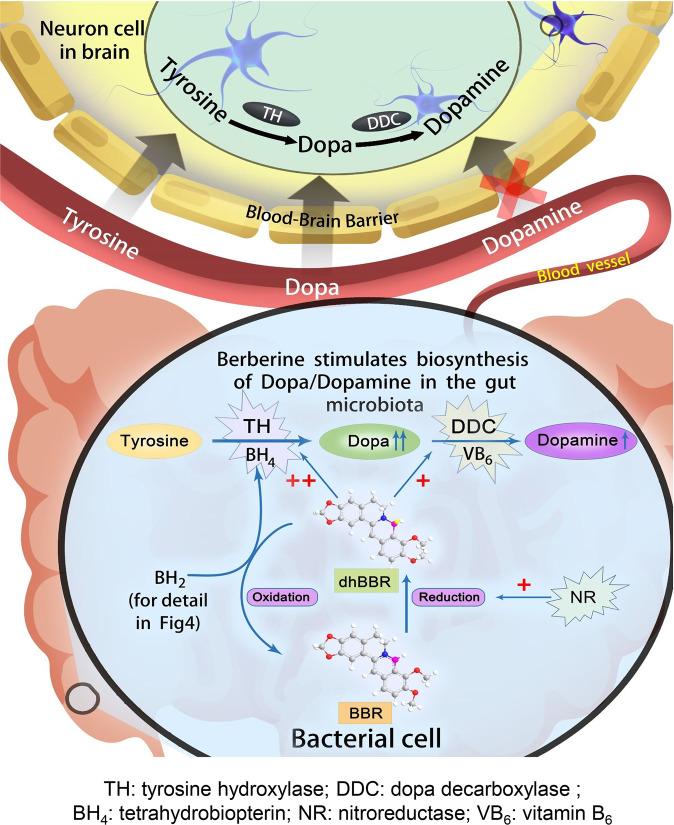
Dopa/dopamine might be the chemical link for the crosstalk between gut and brain. The regulation of Phe–Tyr–dopa–dopamine biosynthesis in the gut microbiota by BBR could improve brain function

Dopamine is a catecholamine neurotransmitter that plays key roles in motor coordination, as well as motivation, reward, addiction, learning, and memory. Dopaminergic neurons synthesize, secrete, reuptake, and metabolize dopamine using a group of enzymes and transporters. Secreted dopamine interacts with its receptors on postsynaptic neurons and adjusts intracellular signaling pathways.^57^ Currently, five subtypes of dopamine receptors (D_1_–D_5_) are documented. The receptor subtypes have distinct anatomical distributions in different areas of the brain^58^ and might be considered biomarkers for various clusters of dopamine-related functions.^59^ Dopamine deficiency (or loss of dopamine-secreting cells) in CNS disorders, such as depression and PD, correlates with functional decline in memory, cognition, motivation, and motor control.^59^ In fact, during the normal aging process, brain dopamine levels are known to decrease by ~7% per decade,^59^ suggesting an important role of dopamine in brain function. Actually, evidence has linked an increase in dopamine to a positive mood, as well as improved executive function.^60^ The present study identifies the gut microbiota as a novel dopa/dopamine-producing “organ” in addition to the brain and discovers BBR a stimulating agent for dopa/dopamine production in the intestinal bacteria. These results also provided support, in view of the mechanism, for the previous observation that oral administration of BBR showed a positive effect on learning and memory.^61^ It appears that regulating the production of neurotransmitters by the gut microbiota (by drugs, food, or nutrients) might improve brain function (or EQ in humans).

As a dopamine precursor, l-dopa has been used as a first-line drug to treat patients with PD for decades^13,62^ and as a DCC inhibitor carbidopa is used together with l-dopa to increase the blood concentration of l-dopa.^63^ l-Dopa is able to cross the blood–brain barrier and enter brain tissue. The central transformation from l-dopa to dopamine likely takes place at surviving dopaminergic terminals and at serotonergic and adrenergic nerve terminals that contain decarboxylase.^64^ The therapeutic effect of l-dopa comes from restoration of the extracellular dopamine level in the dorsal striatum area, which is deficient in endogenous dopamine as a consequence of the neurodegenerative course in PD.^65^ A recent study showed that l-dopa can also restore the striatal activity and improve decision performance in some older adults.^13,66^ However, many patients develop dyskinesias and motor fluctuations within a few years after l-dopa therapy.^67^ In the late stages of PD, l-dopa might induce nonmotor fluctuations with cognitive dysfunction and neuropsychiatric symptoms.^65^ Several presynaptic mechanism studies showed that some of the adverse events of l-dopa might be linked to the large dopamine swings in the brain, concomitant with the peaks and troughs of plasma l-dopa levels.^67^ In the present study, we showed a moderate and constant increase in the dopa/dopamine concentration in the blood and brain (Fig. 1b, c) in animals orally treated with BBR. This is most likely due to the restricted dopa/dopamine-producing capacity in the gut microbiota, and the steady interaction between BBR and bacteria in the intestine, causing a gentle but stable and continuous rise in dopamine in the brain (compared to l-dopa). It might be an advantage in reducing the chance of adverse effects with long-term therapy. Furthermore, in Fig. 3d, we showed that combining BBR with DDC inhibitor benserazide produced an extra increase in dopa in culture, suggesting a BBR-related drug combination for the treatment of PD in the future.

*Enterococcus* is the host bacteria in the gastrointestinal tract. Belonging to *Enterococcus*, *E. faecium*, *and E. faecalis* strains have been used as probiotics to treat diarrhea, antibiotic-associated diarrhea, or irritable bowel syndrome; to lower cholesterol levels; and to improve host immunity.^21,68–74^ In the present study, we found that *E. faecalis* and *E. faecium* contained TH and DDC activity. In fact, a number of bacteria have several means to complete the action of TH and DDC. Through searching the NCBI database, we found bacteria positive for homologs of the TH gene; for instance, *Ralstonia solanacearu* and *Streptomyces achromogenes* contain TH (GenBank: CAD13865.1 and GenBank: ACN39022.1). Tyrosinase present in bacteria (such as *Pseudomonas* and *Bacillus*) could also convert tyrosine into l-dopa.^75–77^ In fact, after searching the NCBI database for protein functions in *Enterobacter*, we found two protein sequences for hydroxylase and ten for decarboxylase in the *E. faecalis* (coded no. WP_077497585.1, WP_002362599.1, as well as WP_002414512.1, WP_002388973.1, WP_002357922.1, WP_002391204.1, WP_002357417.1, WP_002297874.1, WP_002359573.1, WP_002414999.1, WP_011109458.1, and WP_033072606.1). These results explain that, in our experimental system, dopa/dopamine was readily detected in intestinal bacterial cultures in the presence or absence of BBR. Transplantation of either *E. faecalis* or *E. faecium* into the PGF mice caused the synthesis of dopa/dopamine in the intestinal tract; then, intestinal dopa/dopamine entered the blood, leading to an increase in dopamine in the brain. TMP-TFB-derivatized MALDI-MS imaging of dopamine in ICR mouse brains confirmed that the combination of BBR with either *E. faecalis* or *E. faecium* caused a further increase in brain dopamine and ameliorated PD symptoms in the C57 mouse model. The results suggest that the two bacterial strains might be helpful for the brain function through the action of dopa/dopamine production in intestine, and that drug regulation of this process might further improve brain function.

## Conclusion

This study discovered first that the gut microbiota is a new source of dopa/dopamine in the body, and second, BBR enhanced TH to produce l-dopa by triggering the biosynthesis of BH_4_ in the gut microbiota. As BBR has been an OTC drug for many years, it might have immediately applicable potential in regulating gut–brain dialog and improving brain function in humans.

## Materials and methods

### Chemicals and reagents

BBR, dopamine, and l-dopa were obtained from J&K Scientific Ltd (Beijing, China). 3,4-Dihydroxybenzylamine (as an internal standard, IS) was purchased from Alfa Aesar Chemical Co., Ltd (Ward Hill, MA, USA). Tyrosine, phenylalanine, α-methylphenylalanine, BLMA5 (an inhibitor of TH), and benserazide (an inhibitor of DDC) were obtained from Solarbio Life Science Co., Ltd (Beijing, China). ^15^N-Tyrosine (^15^N-Tyr, purity > 98%) was obtained from Cambridge Isotope Laboratories, Inc. (MA, USA). Cefadroxil, terramycin, and erythromycin were obtained from Solarbio Life Science Co., Ltd (Beijing, China). Carbidopa was from Selleckchem (Selleck Chemicals, Houston, TX, USA). P and MPTP-HCL were from Medchemexpress (MedChem Express LLC, Princeton, NJ, USA). The purity of the standards above was >98% (HPLC). The TH ELISA kit was from Shanghai Bangyi Biotechnology, Ltd (Shanghai, China). The DDC ELISA kit was from Shuangying Biological, Ltd (Shanghai, China). The PAH ELISA kit and BH_4_ ELISA kit were obtained from Shanghai Fusheng, Ltd (Shanghai, China) and Shanghai Jianglai, Ltd (Shanghai, China), respectively. The ROS assay kit was from the Nanjing Jiancheng Bioengineering Institute (Nanjing, China).

### Animals

ICR mice (male, 18–22 g), Sprague–Dawley (SD) rats (male, 180–200 g), and C57BL mice (male, 22 ± 3 g) were supplied by Vital River Laboratory Animal Technology Co., Ltd (Beijing, China), and they were housed in a controlled environment (21 ± 2 °C, 12-h light/dark cycle) with free access to food and water during the acclimatization and study periods in an SPF-grade room. The research was conducted in accordance with the institutional guidelines and ethics, and approved by the Laboratories Institutional Animal Care and Use Committee of the Chinese Academy of Medical Sciences and Peking Union Medical College. The research was conducted in accordance with all guidelines and ethics of the Chinese Council on Animal Care.

### Instruments

A LC/MS^*n*^-IT-TOF from Shimadzu Corporation (Kyoto, Japan) was used to identify the chemical structures of BBR and its metabolites. LC-MS/MS (8050, Shimadzu Corporation, Kyoto, Japan) was used for the analysis and quantification of dopamine, phenylalanine, tyrosine, and l-dopa. LC separation was achieved using an Alltima C_18_ column (5 μ × 150 mm, W. R. Grace & Co, Columbia, USA) maintained at 40 °C. The mobile phase for l-dopa, dopamine, tyrosine, and phenylalanine consisted of water–formic acid (100:0.1, v/v) (A) and acetonitrile (B) with a linear gradient elution (A:B, 0 min, 90:10; 2 min, 90:10; 3 min, 5:95; 5 min, 5:95; 5.01 min, 90:10; 8 min 90:10) at a flow rate of 0.8 mL/min during the whole gradient cycle. Shimadzu LC-MS solution (Version 5.72) was used for data acquisition and processing. For positive ESI analysis, the parameters were as follows: nebulizer gas, 3 L/min; drying gas, 10.0 L/min; interface, −4.5 kV; CID gas, 230 kPa; and DL temperature, heat block temperature were maintained at 200 °C and 250 °C, respectively. Quantification was carried out using multiple reaction monitoring mode. The *m*/*z* transitions were 154.20 → 91.10 (*m*/*z*) for dopamine, 198.15 → 107.05 (*m*/*z*) for l-dopa, 165.85 → 120.20 (*m*/*z*) for phenylalanine, 182.00 → 136.10 (*m*/*z*) for tyrosine, and 108.20 → 91.10 (*m*/*z*) for the IS. The peak areas of those compounds in fluid samples and the IS were recorded, respectively.

Leica CM1950 cryostat microtome (Leica Microsystems, Wetzlar, Germany), rapifleX MALDI-TOF/TOF mass spectrometer (Bruker Daltonik, Bremen, Germany), electrostatic field-assisted matrix automatic sprayer (in-house made), indium tin oxide (ITO) glass slides (Bruker Daltonik, Bremen, Germany), and MTP Slide Adapter II (Bruker Daltonik, Bremen, Germany) were used for TMP-TFB-derivatized MALDI-MS imaging of dopamine in ICR mouse brains.

### Quantitative analysis of dopa and dopamine in blood, brain, and feces of ICR mice treated with oral BBR by LC-MS/MS

All animals for the experiment were fasted for 12 h before the test. A total of 60 male ICR mice (19–21 g) were randomly separated into 12 groups (*n* = 5) and BBR was administrated via the oral route. Among them, 20 mice were treated with saline as control, 20 mice were treated with BBR at the dose of 100 mg/kg, and the other 20 mice were treated with BBR at a dose of 200 mg/kg. The samples of blood, brains, and feces were obtained from each group at 0, 6, 12, and 24 h after BBR or saline administration (*n* = 5). With heparin sodium, the blood was centrifuged at 2.4 × 10^3^ × *g* for 5 min. Brains were homogenized with normal saline at a ratio of weight (g)/volume (mL) (1:4). The IS solution (benzylamine, 0.1 µg/mL, 10 µL) was added to the blood or brain homogenate (100 μL). After precipitating protein with acetonitrile (300 µL), samples were mixed for 30 s and centrifuged at 21.1 × 10^3^ × *g* for 10 min. An aliquot (5 µL) was injected into the LC-MS/MS 8050 for analysis. Fecal samples (0.2 g) were extracted with acetonitrile (900 µL) by ultrasonication for 5 min, and then the IS solution (0.1 µg/mL, 100 µL) was added. After centrifugation at 21.1 × 10^3^ × *g* for 10 min, an aliquot (5 µL) was injected into the LC-MS/MS 8050 for analysis. A series of working solutions of dopamine and l-dopa were prepared (at concentrations of 10,000, 1000, 100, 10, 1, 0.2, and 0.1 ng/mL) by diluting the stock solution (1 mg/mL) with water. An aliquot of the solutions above (5 μL) was injected into the LC-MS/MS 8050 for analysis.

### Levels of dopa and dopamine in ICR mice treated with BBR by intraperitoneal administration

BBR (10 and 20 mg/kg) was given to 60 male (19–21 g) ICR mice through the i.p. administration route, and 10 mice were injected with the same amount of normal saline as the control. The blood, brains, and feces were obtained at 0, 6, 12, and 24 h post BBR treatment, as described above. The sample processing procedures were also identical to the former methods. Levels of l-dopa and dopamine in the blood, brains, and feces were analyzed by the method described above.

### Pseudo-germ-free ICR mice treated with BBR

Forty-six ICR mice (male, 19–21 g) were orally treated with cefadroxil (100 mg/kg), terramycin (300 mg/kg), and erythromycin (300 mg/kg) twice a day for 3 days, and pharmacokinetic examination was performed on the third day after the final administration of antibiotics. The colon contents of six mice were also collected on the third day after the final treatment with antibiotics, and the PGF status was confirmed by culturing fecal samples in the anaerobic incubator on a nutrient agar culture medium. Fecal samples from six nonantibiotic-exposed ICR mice served as control samples. Before oral administration of a single dose of BBR (200 mg/kg), the other 40 PGF mice were fasted overnight with free access to water. Blood samples were collected from the posterior orbital venous plexus into a heparinized tube at 0, 6, 12, and 24 h post BBR treatment, and then subjected to the procedure described above. Fecal and brain samples were also collected at 0, 6, 12, and 24 h post BBR treatment. The samples were stored at −20 °C for further analysis. The blood, brain, and fecal samples processing procedures and analyzing methods for dopa or dopamine are described in the former section.

### Determination of ^15^N-dopamine in brain after injection into the mouse colon with ^15^N-tyrosine

^15^N-Tyr (purity > 98%) was supplied by Cambridge Isotope Laboratories, Inc. (MA, USA). Thirty-two ICR mice (male, 19–22 g) were randomly divided into three groups: mice treated with ^15^N-Tyr as group 1 (*n* = 8), the PGF mice treated with BBR and ^15^N-Tyr as group 2 (*n* = 8), and mice treated with BBR and ^15^N-Tyr as group 3 (*n* = 8). The mice in group 2 were given cefadroxil (100 mg/kg), terramycin (300 mg/kg), and erythromycin (300 mg/kg) twice a day for 3 days, and then BBR (200 mg/kg) was given orally to the two groups for three successive days. After the above treatment, ^15^N-Tyr (10 mg/kg) was injected into the colon of anesthetized mice for groups 1–3. After 6 h of administration, the mice were killed, and the brains were collected for the next analysis.

To estimate the dopamine production by the gut microbiota, 12 ICR mice (male, 19–22 g) were randomly divided into 2 groups: mice treated with ^15^N-Tyr as group 1 (*n* = 6), and those treated with antibiotics and ^15^N-Tyr group as group 2 (*n* = 6). The mice in group 2 were given cefadroxil (100 mg/kg), terramycin (300 mg/kg), and erythromycin (300 mg/kg) twice a day for 3 days. Then, ^15^N-Tyr (10 mg/kg) was injected into mice colon under the conditions of anesthesia for both groups. Six hours later, the mice were killed and the brains were collected for analysis.

^15^N-Dopamine was detected by LC-MS/MS 8050, and 155.20 → 137.05 (*m*/*z*) was chosen as the quantitative ion pair for ^15^N-dopamine. The flow speed was set as 0.5 mL/min, and the other analytical conditions of HPLC-MS/MS were the same as those of dopamine. Multiple mass spectrometer analysis was performed by LC-MS^*n*^-IT-TOF. The chromatography conditions were the same as those of LC-MS/MS 8050. Both the temperature of the interface and the curved desolvation line were set at 200 °C. The detector voltage was 1.65 kV, and the collision energy was set as 50%.

### Biotransformation of ^15^N-tyrosine into ^15^N-dopamine in vitro

Blood, liver homogenate, brain homogenate, and intestinal bacteria were used to evaluate the biotransformation of ^15^N-Tyr into ^15^N-dopamine in vitro. ^15^N-Tyr (final concentration 10 μg/mL) was added to the above incubation system. BBR (1 mg/mL, 10 μL) was added to the above incubation system, and methanol (10 μL) was added to the other group as the control group. After incubating for 2, 4, and 6 h, ^15^N-dopamine was tested by LC-MS/MS using the method described above.

### Production of dopa and dopamine stimulated by BBR in the intestinal bacteria in vitro

Colon contents from 15 male ICR mice or 6 SD rats were pooled, and the mixture of colon contents (5 g) was transferred into a flask containing normal saline (100 mL). After mixing thoroughly, the cultures were preincubated under anaerobic conditions with N_2_ atmosphere at 37 °C for 60 min. BBR (10 µL) at different concentrations was added to the rat intestinal bacteria cultures (990 μL), with methanol (10 μL) as the negative control. The final concentrations of BBR in the incubation system were 10 and 20 µg/mL. The cultures were incubated for 0, 6, 12, and 24 h at 37 °C. After termination of the reaction with acetonitrile (1 mL), 10 µL of IS solution (100 ng/mL) was added, and then the incubation was mixed for 30 s and centrifuged at 21.1 × 10^3^ × *g* for 10 min. The supernatant (100 µL) was transferred into an eppendorf tube and dried under nitrogen flow at 37 °C, and then the residue was reconstituted with 100 μL acetonitrile. After centrifugation at 21.1 × 10^3^ × *g* for 5 min, the supernatant was filtered with a 0.22 µm filter membrane, and then 5 µL of the aliquot was injected into the LC-MS/MS 8050 for dopamine and dopa analysis.

### Dopa and dopamine in ten single strains of intestinal bacteria treated with BBR in vitro

Ten strains of intestinal bacteria (*Acinetobacter baumanii* (*A. baumannii*, 1), *Pseudomonas aeruginosa* (*P. aeruginosa*, 2), *Staphylococcus aureus* (*S. aureus*, 3) *E. faecalis* (*E. faecalis*, 4), *E. faecium* (*E. faecium*, 5), *E. coli* (*E. coli*, 6), *P. mirabilis* (*P. mirabilis*, 7), *S. epidermidis* (*S. epidermidis*, 8), *L. acidophilus* (*L. acidophilus*, 9), and *Bifidobacterium breve (B. breve*, 10)) were cultured overnight in the appropriate medium. These bacteria were diluted to a final concentration of 3 × 10^8^ CFU/mL and incubated with blank solvent (methanol), BBR (10 μg/mL), and BBR (20 μg/mL) for 12 h. Then, the contents of dopa and dopamine were analyzed via LC-MS/MS 8050. The sample processing procedures were the same as above. Four kinds of single intestinal bacteria (*E. faecalis*, *E. faecium*, *E. coli,* and *P. mirabilis*) were selected from the ten kinds of single strains above and incubated with BBR (10 μg/mL) for 6, 12, and 24 h. Then the levels of dopa and dopamine were determined quantitatively by LC-MS/MS 8050. The sample processing procedures were the same as above.

### Enzyme activity test of TH and DDC in the bacterial strains of *E. faecalis* and *E. faecium*

*E. faecalis* and *E. faecium* were identically cultivated and counted as described above, and then the bacterial protein extract was prepared as previously described. There were three groups of enzymatic activity tests, including the medium control (group 1), the *E. faecium* group (group 2), and the *E. faecalis* group (group 3). Finally, the detection of enzyme activity was performed according to the manufacturer’s guidelines of the ELISA kit.

### Dopa and dopamine in brain, liver, and small intestine of the ICR mice when incubated with BBR in vitro

After fasting for 12 h, 24 male ICR mice (19–21 g) were sacrificed by cervical dislocation for the collection of brain, liver, and small intestine after rinsing the intestinal contents with saline. Tissue samples were pooled. After weighing, they were homogenized with four volumes (*V* (μL)/*W* (g)) of saline and stored at −80 °C. Ten microliters of BBR at different concentrations was added to the mouse brain, liver, or small intestine homogenate (990 μL), and methanol (10 μL) was added as a negative control. The final concentrations of BBR in the incubation system were 10 and 20 µg/mL. The systems were incubated for 6, 12, and 24 h at 37 °C. Aliquots (100 μL) were removed and quenched in twice the volume of acetonitrile. After adding 10 µL of IS solution (3,4-dihydroxybenzylamine, 100 ng/mL), the samples were mixed for 30 s and centrifuged at 21.1 × 10^3^ × *g* for 10 min. The supernatant was filtered through a 0.22-µm micropore membrane, and a 5 µL aliquot was analyzed by LC-MS/MS 8050 for dopamine or dopa detection. A 1 mg/mL stock solution of dopamine or l-dopa was prepared by dissolving in methanol and was stored at 4 °C. Working solutions were prepared by diluting the stock solution with methanol (to a series of concentrations of 1, 2, 10, 100, 1000, and 10,000 ng/mL of dopamine or l-dopa). A solution containing IS (3,4-dihydroxybenzylamine, 10 μg/mL) was prepared in methanol. Calibration curve samples were prepared by spiking blank samples (distilled water) with 1/100 volume of the working solutions. For dopamine and dopa detection, the final concentrations were 0.1, 0.2 1.0, 10, 100, and 1000 ng/mL. According to the method mentioned above, twice the volume of acetonitrile and IS solution (10 µL) were added to each sample.

### Bacterial composition analysis

The fecal samples of ICR mice for the bacterial composition analysis were classified into three groups: group 1 treated with BBR by oral administration (200 mg/kg, 24 h, *n* = 3), group 2 treated with BBR by i.p. injection (20 mg/kg, 24 h, *n* = 3), and the untreated control group (*n* = 3) as group 3. Gut bacterial composition in the ICR mice was analyzed via 16 S rRNA gene analysis. The 16 S rRNA genes were amplified using the specific primer for 16 S V3–V4: 340F-805R to target the V3–V4 regions of 16 S rRNA. PCR products were mixed in equidensity ratios. Then, a mixture of PCR products was purified with the GeneJET Gel Extraction Kit (QIAGEN, Germany). Sequencing libraries were generated using the NEXTflex Rapid DNA-seq kit for Illumina (New England Biolabs, USA), following the manufacturer’s recommendations, and index codes were added. The library quality was assessed on the Qubit 2.0 Fluorometer (Thermo Scientific, USA) and Agilent Bioanalyzer 2100 system (Agilent Technologies, USA). Finally, the library was sequenced on the HiSeq2500 (Illumina, USA) platform, and 250-bp paired-end reads were generated. Sequences were analyzed using the QIIME software package (Quantitative Insights Into Microbial Ecology). First, the reads were filtered by QIIME quality filters. Then, we picked the representative sequences for each OTU (operational taxonomic unit) and used the RDP (ribosomal database project) classifier to annotate taxonomic information for each representative sequence. Sequences with a similarity over 97% were assigned to the same OTU.

### TH and DDC enzyme activity assays in the intestinal bacteria in vitro

There were three groups of enzymatic activity tests of TH and DDC, including the blank control (group 1), the group treated with BBR (10 µg/mL, group 2), and the group treated with BBR (10 µg/mL) and inhibitors (group 3). BLMA5 hydrochloride (an inhibitor of TH with a final concentration of 100 µM) and benserazide (an inhibitor of DDC with a final concentration of 100 µM) were preincubated with SD rat gut bacterial cultures for 2 h at 37 °C, while the same amount of methanol was added as the control. Then, BBR was added (with a final concentration of 10 µg/mL). After 12 h of incubation, the SD gut bacterial culture (50 mL) was centrifuged at 6.2 × 10^3^ × *g* at 4 °C (3K15 Refrigerated Centrifuge, Sigma Laborzentrifugen GmbH, Osterode am Harz, Germany) for 10 min to remove the culture medium. Then, the residue was reconstituted with PBS and centrifuged under the same conditions. Next, the residue was dissolved in PBS (4 mL) to extract the bacterial proteins with an ultrasonic cell disruptor (Scientz-IID Noise Isolating Chamber, Ningbo Scientz Biotechnology Co. LTD, Ningbo, China) with a circle of 8 s (ultra for 3 s), and the extraction period lasted for 2 h. The protein extracts were centrifuged under the same conditions (see above) to remove insoluble substances. Finally, according to the manufacturer’s guidelines, the detection of enzyme activity of TH and DDC was performed using the TH ELISA kit (Batch number: JL18430) and DDC ELISA kit (Batch number: JL47021) all obtained from Shanghai Jianglai Industrial Limited By Share Ltd (Shanghai, China). The experiment procedure is described in the User Instruction of the kit.

In addition, BLMA5 hydrochloride (100 µM) and benserazide (100 µM) were incubated with SD rat gut bacterial cultures for 12 h at 37 °C, with the same amount of methanol as control. After 12 h incubation, the SD gut bacterial culture (50 mL) was centrifuged at 6.2 × 10^3^ × *g* at 4 °C for 10 min to remove the culture medium, then the residue was reconstituted in PBS, and centrifuged under the same condition. Next, the residue was dissolved in 4 mL PBS to extract the bacterial protein with an ultrasonic cell disruptor. Finally, the enzyme detection for TH and DDC was performed, using the TH ELISA kit and DDC ELISA kit.

### Benzylhydrazine and BLMA5 inhibit intestinal bacteria in vitro

The intestinal contents of three male SD rats (180–200 g) were collected and added to 20 mL of sterilized anaerobic medium per gram of intestinal contents. After mixing and filtering, the intestinal contents were incubated for 1 h at 37 °C, and the corresponding concentration of benzylhydrazine was added at final concentrations of 0, 50, and 100 µM. The samples were incubated for 12 h at 37 °C under anaerobic conditions. Each sample was diluted by 10^3^, 10^4^, and 10^5^-fold. Then, these samples were coated on nutrient agar plates and cultured at 37 °C overnight. The colonies were counted and calculated according to the dilution factor. The experimental procedure to test the effect of BLMA5 on inhibiting the growth of the intestinal bacteria in vitro was consistent with the above.

### Tests of tetrahydrobiopterin and vitamin B_6_

Colon contents from six male SD (180–200 g) rats were pooled, and 5 g of the mixture of colon contents was transferred into a flask containing normal saline (100 mL). After mixing thoroughly, the cultures were preincubated under anaerobic conditions with N_2_ atmosphere at 37 °C for 60 min. BBR (10 µL) at different concentrations was added to the rat intestinal bacteria cultures (990 μL), with methanol (10 μL) as the negative control. The final concentrations of BBR in the incubation system were 10 and 20 µg/mL, respectively. The cultures were incubated for 6, 12, and 24 h at 37 °C. BH_4_ and VB_6_ in the gut bacteria were determined by the BH_4_ ELISA kit and VB_6_ ELISA kit, respectively. Both of them were obtained from Shanghai Jianglai Ltd (Shanghai, China).

### Effects of TH or DDC inhibitors on the production of dopa/dopamine in gut bacteria in vitro

BLMA5 hydrochloride (an inhibitor of TH with a final concentration of 100 µM) and benserazide (an inhibitor of DDC at a final concentration of 100 µM) were preincubated with SD rat gut bacterial cultures for 2 h at 37 °C, while the same amount of methanol was added as the control. Then, BBR was added (with a final concentration of 10 or 20 µg/mL). The incubation times were 6 and 12 h. After termination with acetonitrile, the levels of dopa and dopamine in the incubation were analyzed by the method described above.

### Dopa/dopamine, TH/DDC, and BH_4_/VB_6_ in the brain homogenate treated with BBR or dhBBR

After fasting for 12 h, 20 male ICR mice (19–21 g) were sacrificed by cervical dislocation for brain collection. Brain samples were pooled. After weighing, they were homogenized in saline (with *V* (mL)/*W* (g) = 1:5). Five microliters of BBR or dhBBR at different concentrations was added to the mouse brain homogenate (495 μL), with methanol (5 μL) as the negative control. The final concentrations of BBR or dhBBR in the incubation system were 10 and 20 µg/mL, respectively. The systems were incubated at 37 °C for 6 h. The determination of dopa and dopamine was the same LC-MS/MS method as described above. DDC, TH, BH_4_, and VB_6_ in the brain homogenate were determined by the corresponding ELISA kits. All of them were obtained from Shanghai Jianglai Ltd (Shanghai, China).

### Effects of BBR and dhBBR on dopamine levels in mouse dopamine neurons

After the mouse dopamine neuron cells were cultured to stability (cells purchased from Procell Life Science & Technology Co., Ltd), trypsin was added for digestion. Then, the cells were counted and plated in 48-well microplates. Next, the cells were incubated in a 5% CO_2_ and 37 °C cell incubator for 24 h, with the addition of BBR or dhBBR (final concentration of 10 μg/mL) as the treatment group (DMSO as a control). After 6 h of culture, the cells were removed for disruption, and the LC-MS/MS method was used for detection of dopamine levels (same method as above).

### Reduction of BH_2_ by dhBBR in the presence of dihydrofolate reductase

The purified enzyme reaction system consisted of 25 mM Tris-HCl (pH = 7.4), 1 mM MgCl_2_, 0.1 μ/mL recombinant human DHFR (ProSpec-Tany TechnoGene Ltd, Ness-Ziona, Israel), 1 mM dhBBR (in DMSO), and 1 mM BH_2_ in a final volume of 100 μL. DMSO was added as a control. The DHFR was preincubated via centrifugation at 850 r.p.m. at 37 °C for 3 min, and this step was followed by the addition of NADPH. After reacting for 1 h, the mixture was terminated by adding a threefold volume of ice-cold acetonitrile. Then, the mixture was centrifuged at 12,000 r.p.m. for 5 min, and the supernatant was injected for BH_2_ or BH_4_ analysis by LC-MS/MS.

### Methotrexate, an inhibitor of DHFR, inhibits dopa/dopamine in gut microbiota

Colon contents from three SD rats were pooled and the mixture of colon contents (5 g) was transferred into a flask containing saline (100 mL). After thoroughly mixing, the cultures were preincubated under anaerobic conditions with N_2_ atmosphere at 37 °C for 60 min. Methotrexate (10 μL), an inhibitor of DHFR,^78^ was added to the rat intestinal bacterial cultures (990 μL), with methanol (10 μL) as the negative control. The final concentrations of methotrexate in the incubation system were 100 µM. The cultures were incubated at 37 °C for 12 h. Then the levels of dopa and dopamine were determined quantitatively by LC-MS/MS 8050.

### Parkinson’s disease behavior test on C57 mice

C57BL mice (male, 22 ± 3 g) were housed at five per cage and were randomly divided into five groups (*n* = 10 in each group): the control group (P, i.p., 200 mg/kg/d) as group 1; the MPTP/P-injected group (MPTP, s.c., 20 mg/kg and P, i.p., 200 mg/kg/d) as group 2;^79^ the MPTP/P-injected and BBR-treated (orally) group (MPTP, *s.c*., 20 mg/kg and P, i.p., 200 mg/kg/d; BBR, oral, 200 mg/kg/d) as group 3; the MPTP/P-injected and BBR-treated (i.p.) group (MPTP, s.c., 20 mg/kg and P, i.p., 200 mg/kg/d; BBR, i.p., 10 mg/kg/d) group as group 4; and the MPTP/P-injected and l-dopa/carbidopa-treated group as the positive control (MPTP, s.c., 20 mg/kg and P, i.p., 200 mg/kg/d; days 2–7, l-dopa, s.c., 10 mg/kg/d and carbidopa, s.c., 1 mg/kg) as group 5. In all the groups, rotarod and vertical pole performance test and CT occurred 1 h later. The BBR treatment started 5 days prior to MPTP/P treatment and lasted throughout the entire experiment, and all five groups were treated with MPTP/P for 7 days. Rotarod performance was measured on a rotating rod (YLS-4C Rotarod, Yiyan Science and Technology Development Co., Ltd, Jinan, China) as the mice walked forward to avoid falling off a continuously rotating cylinder. Mice were tested at a rotational speed of 15 r.p.m. on MPTP/P-treated days 1 and 7. Three trials were measured, and the average time on the rod for each mouse was used for data analysis. Time on the rod was used to assess fine motor coordination and balance. The vertical pole descent test (YLS-Q15 Pole, Yiyan Science and Technology Development Co., Ltd, Jinan, China) was conducted on MPTP/P-treated mice on days 1 and 7. Mice were placed at the top of a coarse, vertical wooden pole in a cage, and allowed to descend. They were first given a practice run and then three test trials. The time to reach the cage bottom was recorded. If the mouse did not descend or dropped or slipped down the pole without climbing, a default value of 60 s was recorded. The descent times of the three tests were averaged. CT was performed in a 1000 mL beaker, as previously described.^80^ Each mouse was placed in a glass beaker. The mice freely moved in the beaker and the times of ipsilateral/contralateral paw touches were recorded for 10 min to monitor the physical coordination of each animal. The results were calculated by the ratio of ipsilateral touches to the total of ipsilateral plus contralateral touches. The test was conducted on MPTP/P-treated mice on days 1 and 7. C57 mice were sacrificed immediately to obtain their blood and brains after the behavioral examination. The sample processing procedures were identical to the former part. Dopa and dopamine of the blood, brains, and feces were analyzed by the method mentioned in the former section.

### Transplantation of *E. faecalis* and *E. faecium* to ICR mice

Forty-two ICR mice (male, 18–22 g) were randomly divided into seven groups: the mice treated with PBS as group 1 (*n* = 6, negative control), the mice treated with PBS as group 2 (*n* = 6, PGF control), the mice treated with BBR (200 mg/kg/d, oral) as the group 3, the mice treated with *E. faecalis* (6 × 10^8^ CFU/d, oral) as group 4, the mice treated with *E. faecalis* and BBR (200 mg/kg/d, oral) as group 5, the mice treated with *E. faecium* (6 × 10^8^ CFU/d, oral) as group 6, and the mice treated with *E. faecium* and BBR as group 7. The mice in groups 2–7 were orally given cefadroxil (100 mg/kg), terramycin (300 mg/kg), and erythromycin (300 mg/kg) twice a day for 3 days, and then BBR (200 mg/kg) or bacteria were given orally to the five groups for three successive days. The PGF status was confirmed by culturing fecal samples in the anaerobic incubator on nutrient agar culture medium, using the method described above. After transplantation, the brain, blood, and feces were collected for dopa and dopamine detection.

To verify the colonization of transplanted bacteria, the number of *E. faecium* in fecal samples was evaluated by quantitative PCR. Firstly, the total nucleic acid in samples was extracted by the Genomic DNA extraction Kit (Tiangen Biochemical Technology Co., LTD, Beijing, China) according to the instructions. Then, qPCR was performed in a total volume of 50 μL, which contained 200 nM primers, 1 μL of DNA template, 25 μL of 2×Taq MaterMix (TaKaRa), and 22 μL of water (TaKaRa). Primers were as following: EF-F, TGCTCCACCGGAAAAAGA (5′–3′); EF-R, CACCAACTAGCTAATGCA (5′–3′). qPCR was performed in the Fluorescence quantitative PCR instrument (ABI 7500, Applied Biosystems, Shanghai, China) following the program: 5 min at 95 °C, 30 cycles of 30 s at 94 °C, 30 s at 55 °C, and 30 s at 72 °C, followed by a cycle of 10 min at 72 °C. The calibration curves of the target gene were obtained from the plasmid with target fragment. And the absolute DNA copies were regarded as the level of *E. faecium* in samples.

### Transplantation of *E. coli* to ICR mice

Eighteen ICR mice (male, 18–22 g) were randomly divided into three groups. The mice were treated with PBS as group 1 (*n* = 6, negative control), the PGF mice were treated with PBS as group 2 (*n* = 6, PGF control), and the PGF mice were treated with *E. coli* as group 3. The mice in groups 2 and 3 were orally given cefadroxil (100 mg/kg), terramycin (300 mg/kg), and erythromycin (300 mg/kg) twice a day for 3 days, and the bacterial solution was orally given for three successive days. After transplantation, the brain, blood, and feces were collected for dopa and dopamine detection.

### Test of reactive oxygen species

The levels of ROS were measured in five groups, namely, the blank control (group 1), the *E. coli* group (group 2), the *E. faecium* group (group 3), the *E. faecalis* group (group 4), and the positive control group (*E. coli* treated with H_2_O_2_, group 5). After 24 h of culture of *E. coli*, *E. faecalis*, and *E. faecium*, the number of bacteria was calibrated for the kit assay. The detection of ROS in bacteria was then performed according to the manufacturer’s guidelines. A ROS assay kit was obtained from Nanjing Jiancheng Bioengineering Institute (Nanjing, China).

### 2,4,6-Trimethyl-pyranylium tetrafluoroborate-derivatized MALDI-MS imaging of dopamine in ICR mouse brain

Eighteen ICR mice (male, 18–22 g) were randomly divided into six groups, namely, groups 1–6. The mice in groups 1 and 2 were treated with PBS, and the mice in group 3 were treated with *E. faecalis* (6 × 10^8^ CFU/d, orally); the mice in group 4 were treated with *E. faecalis* (6 × 10^8^ CFU/d, orally) and BBR (200 mg/kg/d, orally); the mice in group 5 were treated with *E. faecium* (6 × 10^8^ CFU/d, orally); the mice in group 6 were treated with *E. faecium* (6 × 10^8^ CFU/d, orally) and BBR. The mice in groups 2–6 were orally given cefadroxil (100 mg/kg), terramycin (300 mg/kg), and erythromycin (300 mg/kg) twice a day for 3 days, and then BBR or bacteria were orally given to the four groups for three successive days.

The brain tissue was placed on a tin foil container after it was separated and frozen in liquid nitrogen until the tissue turned completely white. Then, the frozen brain tissues were cut using a cryostat microtome (Leica CM1950, Leica Microsystems). The tissue was first kept in the freezing chamber, the temperature of which was −20 °C and equilibrated for 2 h. A layer of water was dripped on the sample holder as a frozen embedding solution, and the striatum sections were cut at a thickness of 12 μm. Tissue sections were transferred with a precooled brush onto conductive ITO glass slides (Bruker). After being dried for 30 min, the samples were stored at −80 °C until analysis.

The derivatization method was adopted from a published protocol with slight modification according to the instrument available (Shariatgorji^45^). TMP-TFB was dissolved in methanol to prepare 8 mg/mL stock solutions. Derivatization was performed using solutions containing 120 mL of the stock solution of TMP-TFB in 6 mL of 70% methanol buffered by 3.5 µL of triethylamine. The derivatization reagent was sprayed over the tissue with a flow rate of 40 L/min. The nozzle temperature of the sprayer was kept at 80 °C. The treated tissue samples were then incubated for 30 min (dried by nitrogen flow every 10 min) in a chamber saturated with 50% methanol solution.

α-Cyano-4-hydroxy-cinnamic acid (CHCA) was used as the assisted matrix. CHCA (10 mg/mL) was prepared in a 50% methanol solution containing 0.1% trifluoroacetic acid. The MALDI-MS matrix was applied to the tissue using the same in-house automatic sprayer. The nozzle temperature was set at 95 °C with a flow rate of 20 L/min. MALDI-MS imaging experiments were performed using a rapifleX MALDI-TOF/TOF mass spectrometer (Bruker). The coated tissue slice was placed in the mass spectrometer. After TeachMaker calibration, the pixel size of the imaging resolution was defined for optimal acquisition performance. MS imaging data were visualized using FlexImaging (Bruker). After the data were normalized by total ion current, the neurotransmitter peak was determined, and the mass spectrum peak after derivatization was 444.1985 ± 0.02 to evaluate the distribution of dopamine in the tissue area. The peak height was selected to represent the relative abundance of biomolecules. The colored spots in the image represent the location of the compound, and the color of each spot is related to the intensity of the signal detected by the laser at each point or pixel.

### Parkinson’s disease behavior test on C57 mice treated with *E. faecalis* and *E. faecium*

C57BL mice (male, 22 ± 3 g) were housed at four to five per cage and were randomly divided into seven groups (*n* = 10–13 in each group): the control group (P, i.p., 200 mg/kg/d) as group 1; the MPTP/P-injected group (MPTP, s.c., 20 mg/kg and P, i.p., 200 mg/kg/d) as group 2; the MPTP/P-injected and BBR-treated (orally) group (MPTP, s.c., 20 mg/kg and P, i.p., 200 mg/kg/d; BBR, orally, 200 mg/kg/d) as group 3; the MPTP/P-injected and *E. faecium*-treated (orally) group (MPTP, s.c., 20 mg/kg and P, i.p., 200 mg/kg/d; *E. faecium*, orally, 6 × 10^8^ CFU/d) group as group 4; the MPTP/P-injected and *E. faecalis*-treated (orally) group (MPTP, s.c., 20 mg/kg and P, i.p., 200 mg/kg/d; *E. faecalis*, orally, 6 × 10^8^ CFU/d) as group 5; the MPTP/P-injected and *E. faecium* and BBR-treated (orally) group (MPTP, s.c., 20 mg/kg and P, i.p., 200 mg/kg/d; *E. faecium*, orally, 6 × 10^8^ CFU/d; BBR, orally, 200 mg/kg/d) group as group 6; and the MPTP/P-injected and *E. faecalis* and BBR-treated (orally) group (MPTP, s.c., 20 mg/kg and P, i.p., 200 mg/kg/d; *E. faecalis*, orally, 6 × 10^8^ CFU/d; BBR, orally, 200 mg/kg/d) as group 7. In all the groups, the rotarod and vertical pole performance were tested 1 h later. The treatment of BBR, *E. faecium*, or *E. faecalis* started 5 days prior to MPTP/P treatment and lasted throughout the entire experiment, and all seven groups were treated with MPTP/P for 7 days. The tests of rotarod performance and vertical pole descent were performed as previously described on days 3, 5, and 7. Three trials were measured, and the average time for each mouse was used for data analysis. All C57 mice were sacrificed immediately to obtain their blood and brains after behavioral examination. The sample processing procedures were also identical to the former part. The levels of dopa/dopamine in blood, brains, and feces were analyzed by the method mentioned in the previous section.

### Clinical trials

Twenty-eight patients (17 males, 11 females; age 65.2 ± 7.0) with high levels of blood lipids/glucose (blood cholesterol (mmol/L), 5.56 ± 1.03; triglyceride (mmol/L), 3.17 ± 4.10; LDL-C (mmol/L), 3.10 ± 0.81; and FBG (mmol/L), 6.76 ± 2.65) were enrolled in the study in the Outpatient Section of the First Hospital of Jilin University in Changchun in the summer of 2017. The study was approved by the institutional ethics committee of the hospital (ChiCTR-OPN-17012942), and all the patients provided informed consent. Seven of them had both high blood lipids and glucose. The subjects were not undergoing any drug treatment before enrollment. Before BBR treatment, blood samples were taken from each individual as the control; 5-gram fecal samples from the 28 individuals were collected for enzyme activity tests (TH and DDC) immediately.

The 28 patients were orally treated with BBR (0.5 grams, bid) for 2 months. Blood samples were collected in the morning on the first day 2 months later and placed at −20 °C. Before the last treatment (within 24 h), 5-gram fecal samples from the 28 individuals were collected, and immediately prepared for measurement of the enzyme activity of TH and DDC. The fecal samples of BBR treatment were also used for the bacterial composition analysis and calculation of the relative abundance of Enterococcaceae.

The concentrations of dopa and dopamine in plasma and feces of the patients (before/after BBR treatment) were measured using LC-MS/MS as described.

### Statistical analysis

The statistical analyses were conducted using two-way ANOVA and Student’s *t* test with GraphPad Prism Version 5 (GraphPad Software, CA, USA). The data are expressed as the means ± standard deviation. *P* values <0.05 were considered statistically significant.

## Supplementary information

Supplementary Materials for Oral berberine improves brain dopa/dopamine levels to ameliorate Parkinson’s disease by regulating gut microbiota

## Data Availability

The data used and/or analyzed to support the findings of this study are available in this paper or the Supplementary Information. Any other raw data that support the findings of this study are available from the corresponding author upon reasonable request.
