# Supplementary material for: Oral berberine improves brain dopa/dopamine levels to ameliorate Parkinson’s disease by regulating gut microbiota
Source: Signal Transduct Target Ther. 2021 Feb 24;6:77. doi: 10.1038/s41392-020-00456-5 (PMC7902645; doi:10.1038/s41392-020-00456-5)
Supplement: Supplementary file 1 — Supplementary Materials for Oral berberine improves brain dopa/dopamine levels to ameliorate Parkinson’s disease by regulating gut microbiota [file 41392_2020_456_MOESM1_ESM.docx]

**Supplementary Materials for**

**Oral berberine improves brain dopa/dopamine levels to ameliorate Parkinson’s disease by regulating gut microbiota**

Yan Wang^1^*^†^, Qian Tong^2†^, Shu-Rong Ma^1†^, Zhen-Xiong Zhao^1†^, Li-Bin Pan^1^, Lin Cong^1^, Pei Han^1^, Ran Peng^1^, Hang Yu^1^, Yuan Lin^1^, Tian-Le Gao^1^, Jia-Wen Shou^1^, Xiao-Yang Li^1^, Xian-Feng Zhang^2^, Zheng-Wei Zhang^1^, Jie Fu^1^, Bao-Ying Wen^1^, Jin-Bo Yu^1^, Xuetao Cao^3^*, Jian-Dong Jiang^1^*

^†^These authors made an equal contribution to this work.

*Corresponding to: jiang.jdong@163.com; caoxt@immunol.org; [wangyan@imm.ac.cn](mailto:wangyan@imm.ac.cn).

**This PDF file includes:**

Figures S1 to S6

**
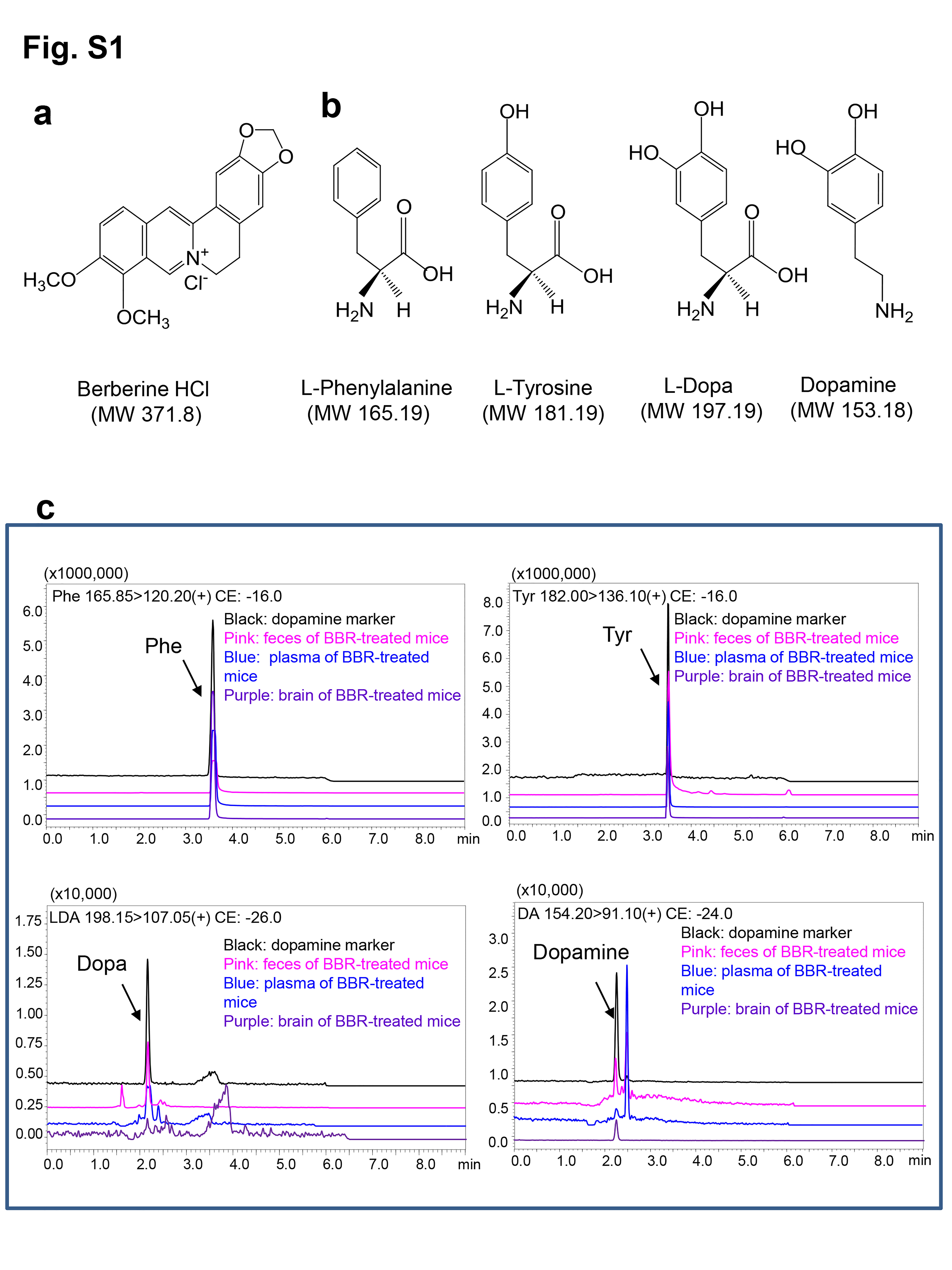
**

**Figure. S1. Chemical structures of the compounds.**

**a** Berberine hydrochloride. **b** *L*-Phenylalanine, *L*-tyrosine, *L*-dopa, and dopamine. **c** Mass chromatograms of *L*-phenylalanine, *L*-tyrosine, *L*-dopa, and dopamine detected in mice brain, plasma and feces.

**
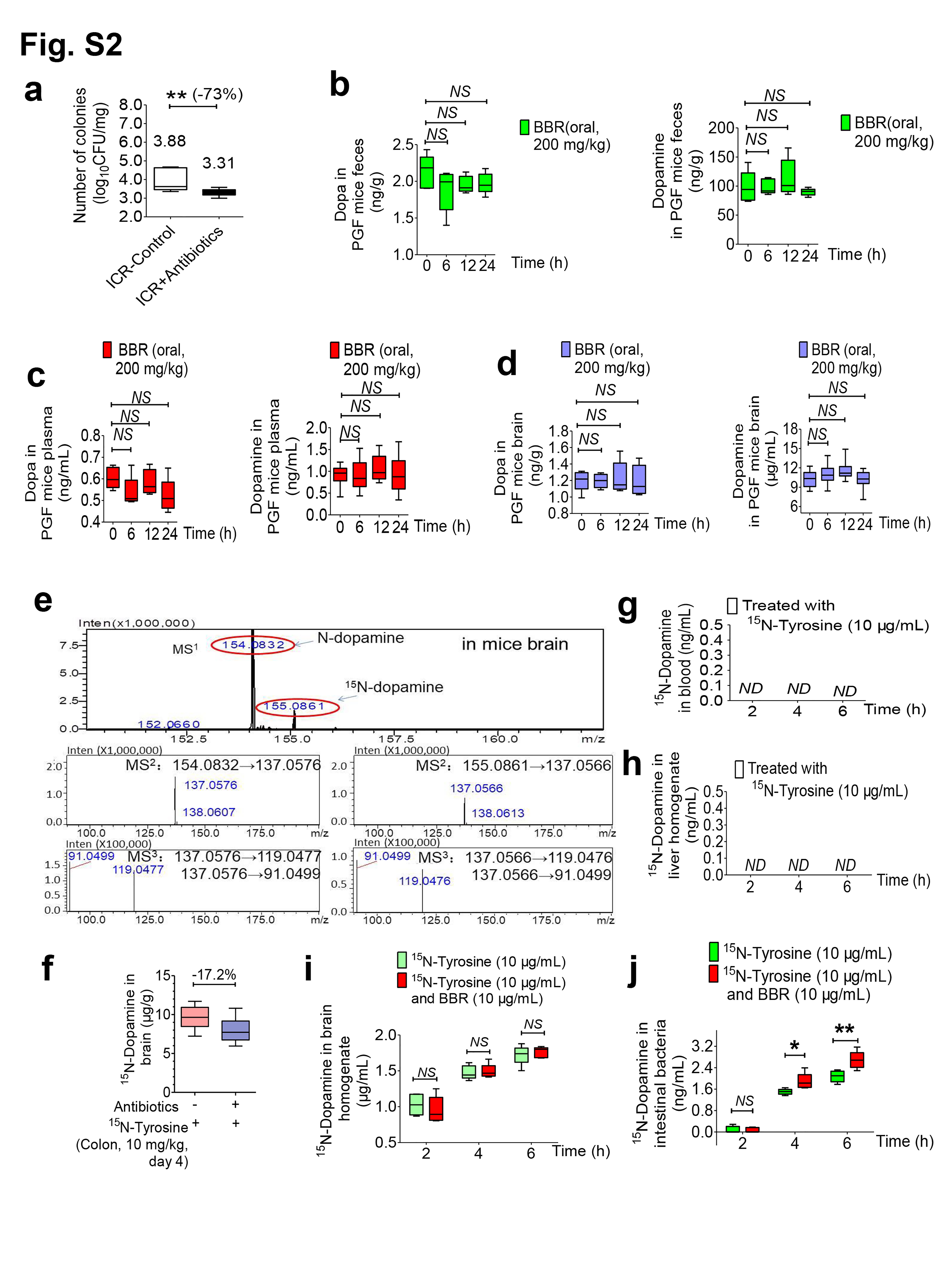
Figure. S2. BBR increased the production of ^15^N-dopamine in brain and negative results were observed in PGF mice**.

**a** Number of colonies in the PGF mice was significantly lower (-73%) than that in the normal mice by antibiotics (***P*<0.01). **b-d** BBR did not increase the levels of dopa/dopamine in feces, plasma and brain of PGF mice. **e** Multistage mass spectrum of dopamine and ^15^N-dopmaine. Dopamine: 154.0832 (MS^1^) →137.0576 (MS^2^) and 137.0576→119.0477 (MS^3^), 137.0576→91.0499 (MS^3^); ^15^N-dopmaine: 155.0861 (MS^1^) →137.0566 (MS^2^) and 137.0566→119.0476 (MS^3^), 137.0566→91.0499 (MS^3^). **f** Oral antibiotics without BBR treatment showed that ^15^N-dopamine in brain decreased by -17.2% in the control group of antibiotics. **g-j** ^15^N-dopamine was not detected in blood and liver homogenate after treated with ^15^N-tyrosine (10 μg/mL) for 2, 4 and 6 h. **h** ^15^N-dopamine in brain homogenate was detected after treated with ^15^N-Tyrosine (10 μg/mL) for 2, 4 and 6 h. **i** ^15^N-dopamine in gut microbiota was detected after treated with ^15^N-Tyrosine for 2, 4 and 6 h, and berberine stimulated the gut microbiota to produce ^15^N-dopamine (**P*<0.05 and ***P*<0.01).

**
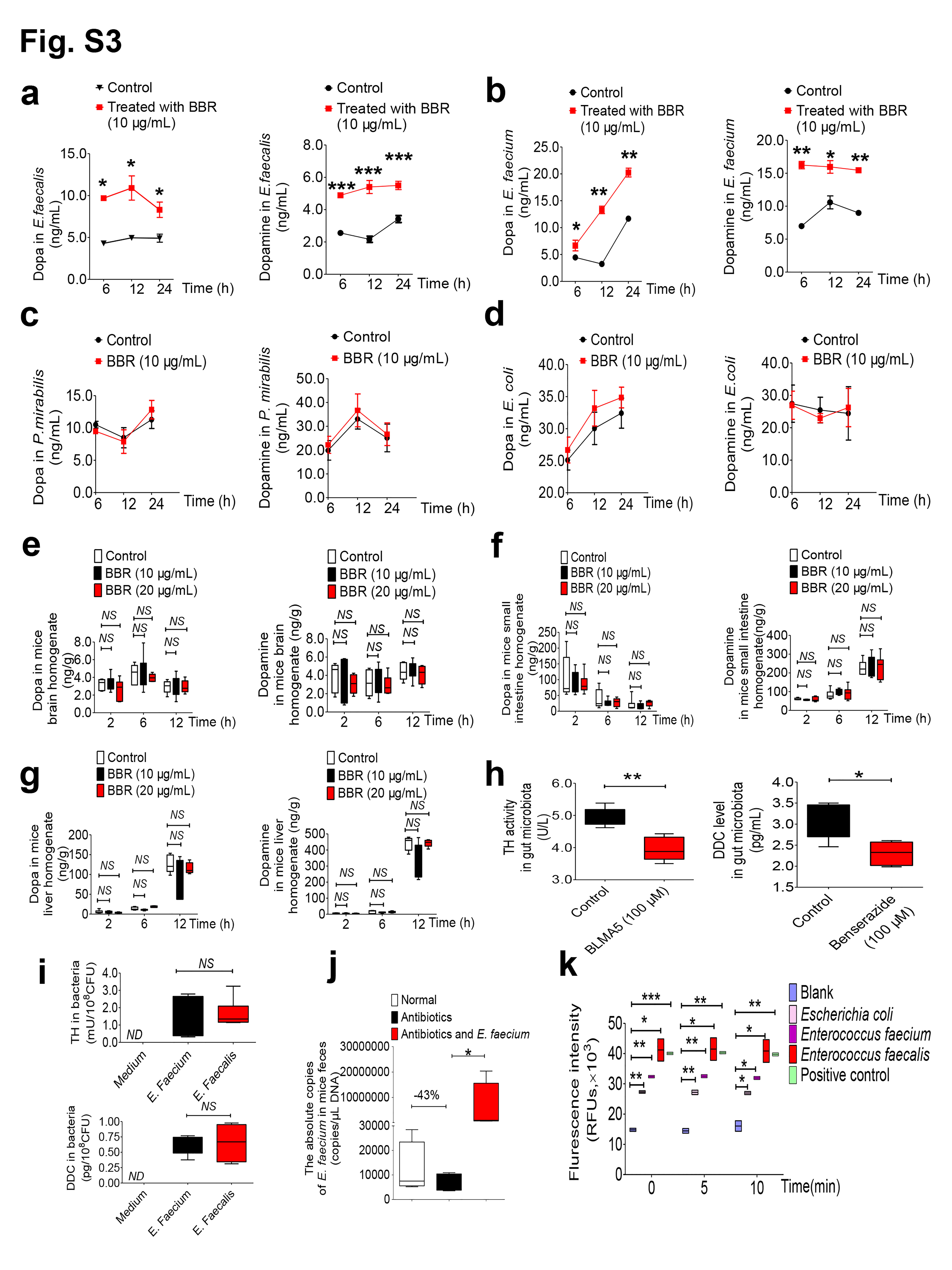
**

**Figure. S3. BBR influenced the levels of dopa/dopamine in *E. faecalis, E. faecium, P. mirabilis* and *E. coli*.**

**a** BBR stimulated *E. faecalis* to produce dopa (**P*<0.05) and dopamine (****P*<0.001) within 24h*.* **b** Dopa (**P*<0.05) and dopamine (****P*<0.001) generated by *E. faecium* could also be induced by BBR with a significant increase. **c** BBR (10 μg/mL) stimulated *P.mirabilis* to produce dopa or dopamine with a slight increase but no significance. **d** BBR did not increase the level of dopa/dopamine in *E. coli* at 6, 12 and 24 h. **e-g** BBR did not increase dopa/dopamine levels in mice brain, small intestine and liver homogenate. **h** The activity of TH decreased after treating the gut bacteria of SD rat with TH inhibitor (BLMA5, 100 μM) for 12 h (***P*<0.01); DDC level decreased after treating the gut bacteria with DDC inhibitor (benserazide, 100 μM) for 12 h (**P*<0.05). **i** Activity of TH and DDC in *E. faecalis* and *E. faecium* were well detected, but they were not detected in medium. **j** The absolute copy numbers of *E. faecium* in mice feces (copies/μL DNA). Copies of *E. faecium* increased significantly in the *E. faecium* transplanted group as compared to that in the antibiotic treated group (**P*<0.05), suggesting that the bacteria have colonized successfully. Additionally, the bacterial DNA copy number in the antibiotics treated mice decreased by 43%, with respect to the normal control. **k** Intensity of flurescence in *E. faecalis, E. faecium* and *E. coli* was measured to assess the existing of reactive oxygen species ROS in the bacterial cell (**P*<0.05, ***P*<0.01, and ****P*<0.001).


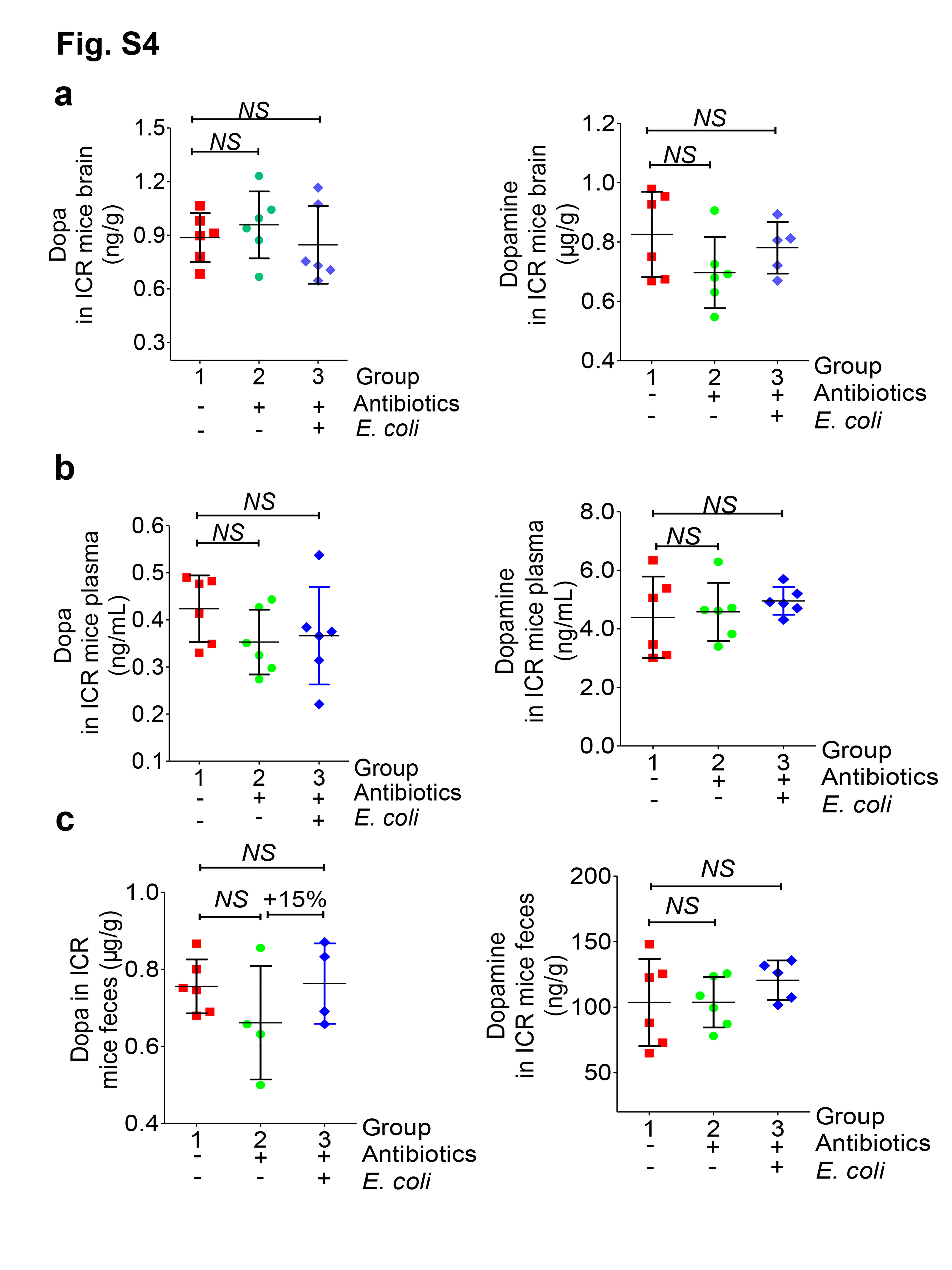


**Figure. S4.** Translation of *E. coli* into the mice treated with antibiotics did not influence the dopa/dopamine levels in the brain of ICR mice (**a**), plasma (**b**) and feces (**c**) (*NS*: no significance).

**
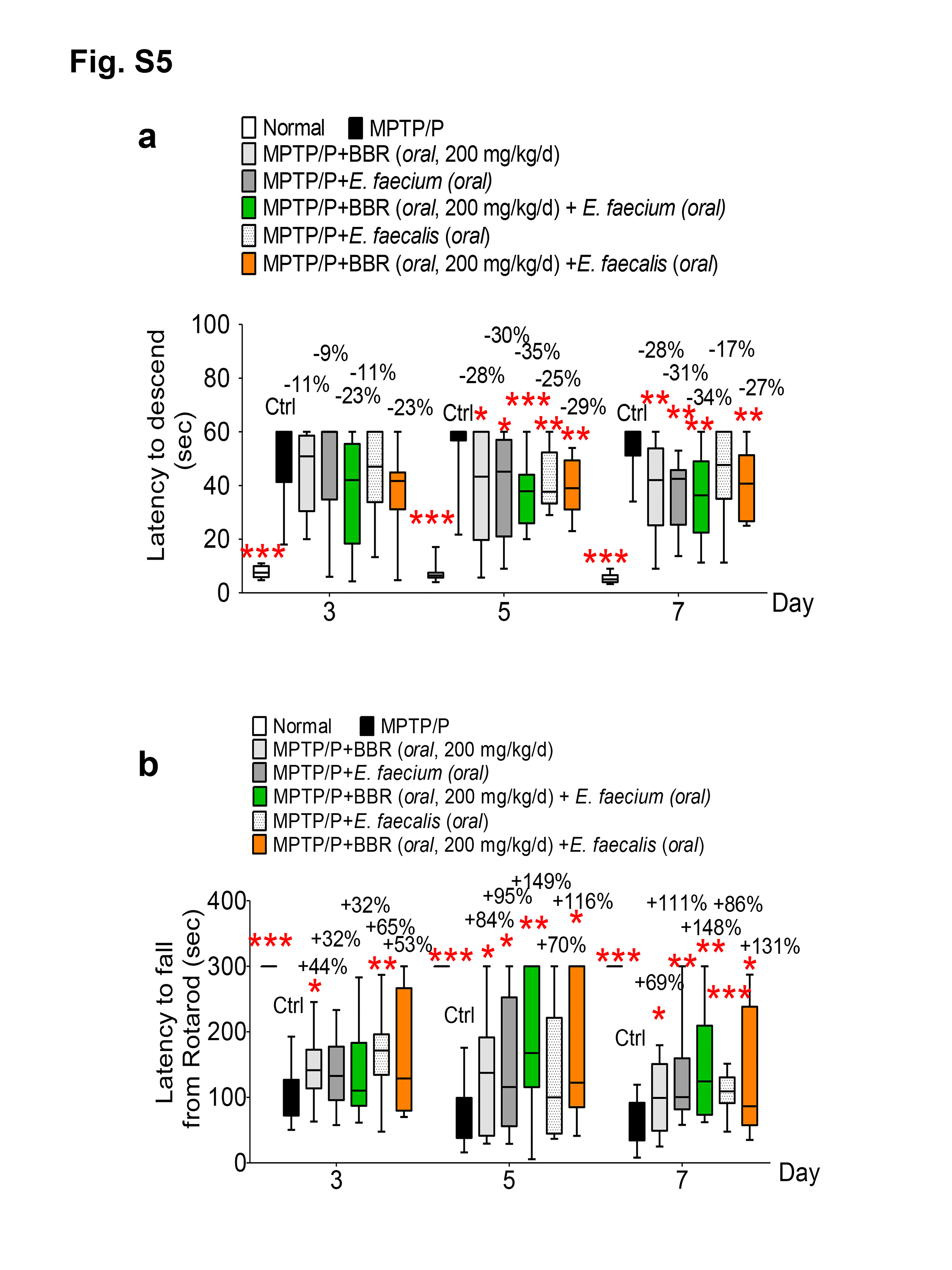
Figure. S5. *E. faecalis* and *E. faecium* improved the brain function of the C57BL mice damaged by MPTP/probenecid in the presence of BBR.**

**a-b** Brain functional test results in the PD mice models. Treating mice with both MPTP (25 mg/kg/d, *s.c.*) and probenecid (P, 250mg/kg/d, *i.p.*) for 7 days successfully generated the damage in brain function, showing a significant increase of Latency to Descent duration (LD, ****P*<0.001) and decrease of Latency to Fall from Rotarod duration (LFR, ****P*<0.001) in the disease model mice. Treating the model mice with BBR (200 mg/kg/d, *oral*) for 3, 5 and 7 days significantly reduced the LD by 11%, 28% and 28% (**P*<0.05, ***P*<0.01) and elevated the LFR by 44%, 84% and 69% (**P*<0.05), respectively. Orally treating the model mice with *E. faecium* significantly reduced the LD by 9%, 30% and 31% (**P*<0.05, ***P*<0.01), and elevated LFR by 32%, 95% and 111% (**P*<0.05, ***P*<0.01) on day 3, 5 and 7, respectively. Likewise, treating the model mice with *E. faecalis* for 3, 5 and 7 days significantly reduced the LD by 11%, 25% and 17% (***P*<0.01) and elevated LFR by 65%, 70% and 86% (***P*<0.01, ****P*<0.001), respectively. BBR might regulate the increase exerted by *E. faecalis* and *E. faecium* (**P*<0.05, ***P*<0.01, and ****P*<0.001).

**
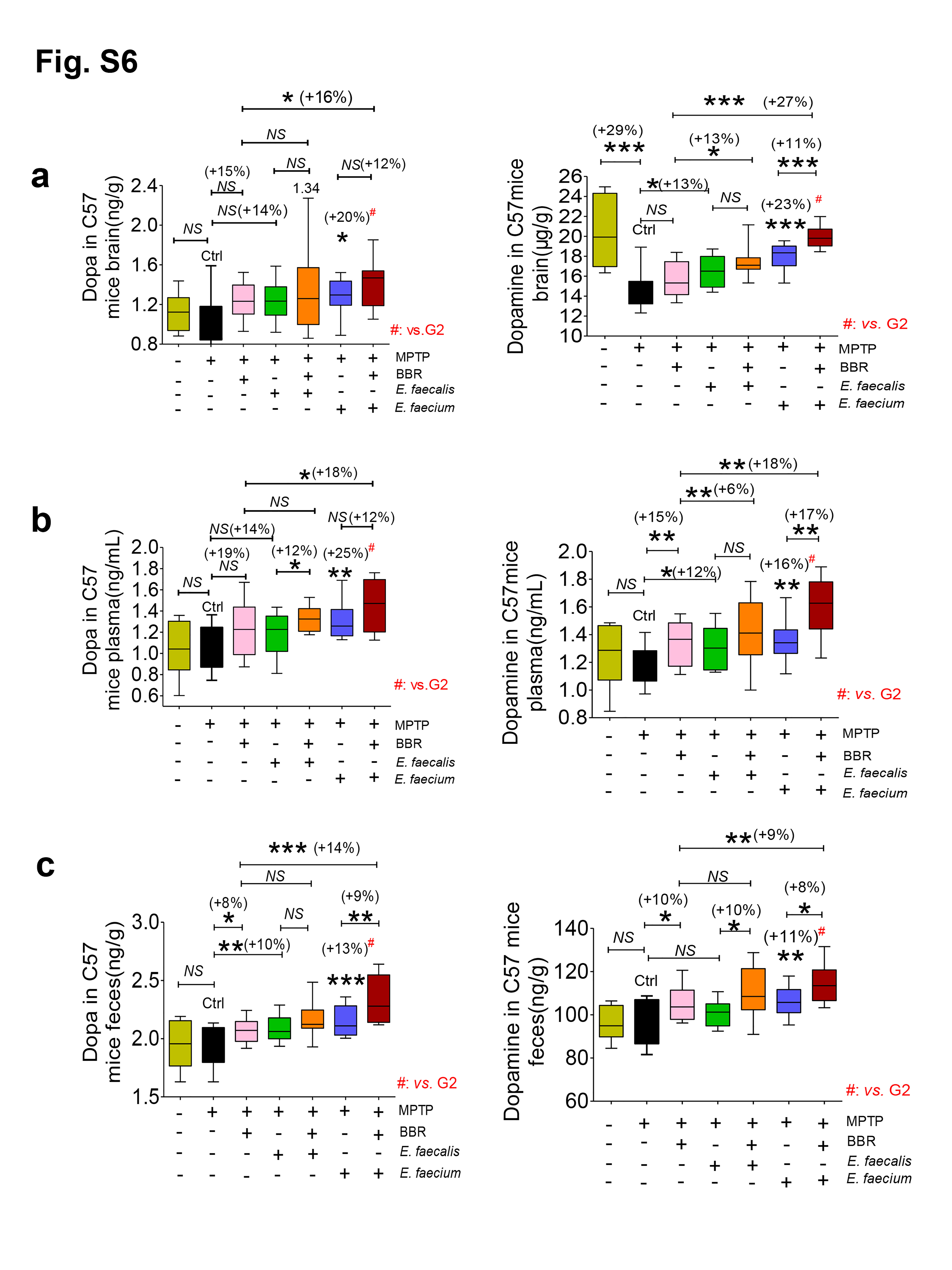
Figure. S6.** *E. faecalis* and *E. faecium* increased the level of dopa and dopamine in C57 mice brain (**a**), plasma (**b**) and feces (**c**) and BBR regulated the increase exerted by *E. faecalis* and *E. faecium* (**P*<0.05, ***P*<0.01, and ****P*<0.001).
